# MAP4K4 and WT1 mediate SOX6‐induced cellular senescence by synergistically activating the ATF2–TGFβ2–Smad2/3 signaling pathway in cervical cancer

**DOI:** 10.1002/1878-0261.13613

**Published:** 2024-02-21

**Authors:** Han Zheng, Mingchen Liu, Shu Shi, Hongxin Huang, Xingwen Yang, Ziheng Luo, Yarong Song, Qiang Xu, Tingting Li, Lixiang Xue, Fengmin Lu, Jie Wang

**Affiliations:** ^1^ Department of Microbiology and Infectious Disease Center, School of Basic Medical Sciences Peking University Health Science Center Beijing China; ^2^ NHC Key Laboratory of Medical Immunology Peking University Beijing China; ^3^ Department of Biomedical Informatics, School of Basic Medical Sciences Peking University Health Science Center Beijing China; ^4^ Department of Radiation Oncology Cancer Center of Peking University Third Hospital, Peking University Third Hospital Beijing China

**Keywords:** cellular senescence, cervical cancer, cisplatin, senolytics, SOX6

## Abstract

SRY‐box transcription factor 6 (*SOX6*) is a member of the SOX gene family and inhibits the proliferation of cervical cancer cells by inducing cell cycle arrest. However, the final cell fate and significance of these cell‐cycle‐arrested cervical cancer cells induced by SOX6 remains unclear. Here, we report that SOX6 inhibits the proliferation of cervical cancer cells by inducing cellular senescence, which is mainly mediated by promoting transforming growth factor beta 2 (*TGFB2*) gene expression and subsequently activating the TGFβ2–Smad2/3–p53–p21^WAF1/CIP1^–Rb pathway. SOX6 promotes *TGFB2* gene expression through the MAP4K4–MAPK (JNK/ERK/p38)–ATF2 and WT1–ATF2 pathways, which is dependent on its high‐mobility group (HMG) domain. In addition, the SOX6‐induced senescent cervical cancer cells are resistant to cisplatin treatment. ABT‐263 (navitoclax) and ABT‐199 (venetoclax), two classic senolytics, can specifically eliminate the SOX6‐induced senescent cervical cancer cells, and thus significantly improve the chemosensitivity of cisplatin‐resistant cervical cancer cells. This study uncovers that the MAP4K4/WT1–ATF2–TGFβ2 axis mediates SOX6‐induced cellular senescence, which is a promising therapeutic target in improving the chemosensitivity of cervical cancer.

## Graphical abstract

MAP4K4 and WT1 contribute to SOX6‐induced cellular senescence in cervical cancer by synergistically activating the ATF2–TGFβ2–Smad2/3 signaling pathway. The SOX6‐induced senescence of cervical cancer cells contributes to resistance to cisplatin treatment. Senolytics can be used to enhance sensitivity to cisplatin treatment by inducing apoptosis of the SOX6‐induced senescent cervical cancer cells.

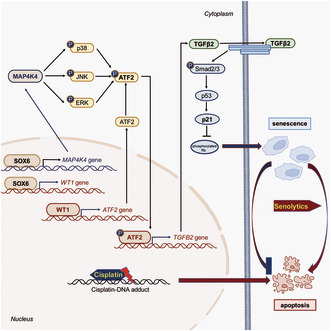

**Abbreviations**
ATF2: activating transcription factor 2
CC: cervical cancer
DEGs: differentially expressed genes
Dox: doxycycline
GO: gene ontology
HCQ: hydroxychloroquine
HMG domain: high‐mobility group domain
hTERT: human telomerase reverse transcriptase
LEF1: lymphoid enhancer‐binding factor 1
MAZ: MYC‐associated zinc finger protein
SAHF: senescence‐associated heterochromatin foci
SASP: senescence‐associated secretory phenotype
SOX6: SRY‐box transcription factor 6
TGFB2: transforming growth factor beta 2
TSS: transcription start site
WT1: WT1 transcription factor

## 1 Introduction

Cervical cancer (CC) is the fourth most common cancer and the fourth leading cause of cancer death in females [1]. The main cause of CC is high‐risk human papillomavirus (HPV) infection, which is responsible for about 90% of CC [2, 3]. Although HPV vaccination and CC screening have achieved initial success, it still needs time to improve the coverage of vaccination and CC screening, especially in low‐income and lower middle‐income countries where CC is more common than high‐income countries [4, 5, 6]. In clinical practice, platinum‐based neoadjuvant chemotherapy followed by surgery and radiotherapy is an effective treatment strategy for locally advanced CC; however, 15–34% of CC patients still have a poor response to the platinum‐based neoadjuvant chemotherapy [7, 8]. Therefore, novel therapeutic targets and strategies are needed for cisplatin‐resistant CC.

It has been reported that E6 and E7 oncoproteins of HPV promote the progress of CC mainly through inhibiting cellular senescence [9]. As we know, cellular senescence is described as an irreversible cell cycle arrest, which is mainly mediated by the p53–p21^WAF1/CIP1^–Rb and p16^INK4a^–Rb pathways [10]. Since senescent cancer cells exhibit cell cycle arrest and can prevent further genomic instability, inducing cellular senescence is becoming a potential therapeutic strategy of cancer therapy [11, 12]. However, senescent cancer cells also exhibit resistance to apoptosis, and the senescence‐associated secretory phenotype (SASP) factors secreted by senescent cells have protumorigenic effects [13]. Therefore, a “one‐two punch” cancer therapy is proposed, which is described as a senescence‐inducing therapy, followed by a kind of drugs that selectively kill senescent cells [14]. These drugs are called senolytics, including Bcl‐2 homology 3 (BH3) domain mimetic drugs ABT‐199 (venetoclax) and ABT‐263 (navitoclax), which have little side effects to normal cells [15].

We have previously found that SOX6 inhibits the proliferation of CC cells by inducing cell cycle arrest [16], and SOX6 is highly expressed in the CC tissues of cisplatin‐resistant patients and reduces the chemotherapy sensitivity of CC [17]. SOX6 is a member of the SOX gene family which encodes proteins that regulate the expression of target genes by binding their highly conserved high‐mobility group (HMG) domain to the transcriptional regulatory region of the target gene [18]. SOX6 is closely related to the development of many tumors, including CC [16, 17, 19], hepatocellular carcinoma [20, 21], osteosarcoma [22], glioblastoma [23], lung adenocarcinoma [24], breast cancer [25], ovarian cancer [26], and so on. In this study, we found that SOX6 could eventually lead to senescence of CC cells after inducing autophagy. Furthermore, we explored the mechanism of SOX6‐induced cellular senescence *in vitro* and *in vivo*. In addition, we explored the role of SOX6‐induced cellular senescence in the treatment of cisplatin and its value in improving the chemotherapy sensitivity of CC by combining with senolytic therapy.

## 2 Materials and methods

### 2.1 Cell lines

The human CC cell lines HeLa (RRID: CVCL_0030), CaSki (RRID: CVCL_1100) and SiHa (RRID: CVCL_0032) used in this study were purchased from the American Type Culture Collection (ATCC, Manassas, VA, USA) and were authenticated by short tandem repeat profiling. Cells were all maintained in Dulbecco's modified eagle medium (DMEM) supplemented with 10% fetal bovine serum (FBS). HeLa‐HA‐SOX6‐tet, HeLa‐HA‐SOX6ΔHMG‐tet, and HeLa‐SOX6KO cells were previously constructed in our laboratory [16, 17]. CaSki‐HA‐SOX6‐tet and SiHa‐HA‐SOX6‐tet cells were constructed in this study and were maintained in DMEM with 10% FBS and 1 μg·mL^−1^ puromycin. All cells were incubated at 37°C with 5% CO_2_ in a humidified incubator. All cell lines used in this research were mycoplasma free.

### 2.2 Plasmids

The plex‐HA‐SOX6 and plex‐HA‐SOX6ΔHMG plasmids were previously constructed in our laboratory [16]. The pGL3‐TGFB2pro‐luc, pGL3‐ATF2pro‐luc, and pGL3‐WT1pro‐luc plasmids were constructed by ligating the PCR products of the promoter region of *TGFB2*, *ATF2*, and *WT1* genes into the pGL3‐Basic plasmid, respectively. The corresponding mutant plasmids were constructed by site‐directed mutagenesis method. The expression plasmids pCDH‐flag‐TGFB2, pCDH‐flag‐ATF2, and pCDH‐flag‐WT1 were constructed by ligating the PCR products of the coding sequences of *TGFB2*, *ATF2*, and *WT1* genes into the pCDH‐Vector plasmid, respectively. The primer sequences used for plasmids construction were shown in Table S1.

### 2.3 RNA interference

*TGFB2*, *MAP4K4*, *ATF2*, and *WT1* gene‐specific siRNAs were transfected into cells using Lipofectamine RNAiMAX (Thermo Fisher Scientific, Waltham, MA, USA). Cells were collected at 3 days post‐transfection, and the efficiency of siRNA was evaluated by RT‐qPCR and western blot.

### 2.4 RNA extraction, reverse transcription, and RT‐qPCR

TRIzol Reagent (Thermo Fisher Scientific) was used for extracting total RNA, and Transcriptor First Strand cDNA Synthesis Kit (Roche, Basel, Kanton Basel, Switzerland) was used for the reverse transcription (RT). Real‐time PCR (qPCR) was performed by Applied Biosystems StepOne plus Real‐Time PCR system (Thermo Fisher Scientific). The primers used for detecting the mRNA levels of *TGFB2*, *WT1*, *ATF2* and *ACTB* genes are listed in Table S2.

### 2.5 Chromatin immunoprecipitation (ChIP) assay

ChIP assays were performed as described previously [17]. In brief, cells were harvested at 48 h post transfection, and were crosslinked by 1% methanal (J&K Scientific, Beijing, China), and then were treated with glycine (FeiMoBio, Beijing, China). Subsequently, cells were lysed with SDS lysis buffer, fragmented by ultrasound and immunoprecipitated with anti HA‐tag (ab9110, Abcam, Cambridge, UK) or IgG (I5006, Sigma‐Aldrich, St. Louis, MO, USA).

### 2.6 Dual‐luciferase assay

Cells were transfected with gene expression plasmid, Firefly luciferase reporter plasmid and PRL‐TK (Renilla luciferase) plasmid using Lipofectamine 2000 (Thermo Fisher Scientific). The activities of Firefly and Renilla luciferases were measured by dual‐luciferase Reporter Assay kit (Promega, Madison, WI, USA) at 48 h post‐transfection.

### 2.7 Cell counting kit‐8 (CCK‐8) assay

In brief, 10 μL CCK‐8 (Solarbio, Beijing, China) was added into 100 μL cell culture supernatant of each well and incubated at 37°C for 1 h. Subsequently, the cell viability was analyzed by measuring the absorbance at 450 nm with Microplate reader (Bio‐Rad, Hercules, CA, USA).

### 2.8 Colony formation assay

Cells were seeded into 6‐well plates (500 cells/well) and incubated for 14 days. Colonies were fixed in 4% paraformaldehyde (PFA) (Solarbio) for 20 min and stained with 1% crystal violet (Solarbio) for 20 min at room temperature, and then the cell colonies were observed.

### 2.9 Soft agar colony formation assay

In brief, 1.2% agarose (Coolaber, Beijing, China) solution and DMEM were mixed with a ratio of 1:1 and added in a 6‐well plate. When the solution was solidified, cells (500 cells/well) in DMEM and 0.7% agarose solution were mixed with a ratio of 1:1, and then the cell mixtures were added into the 6‐well plate and incubated for 14 days.

### 2.10 Flow cytometric analyses

The Flow cytometric analyses for cell cycle and apoptosis detections were performed as described previously [16, 17]. The samples were analyzed by BD FACSCalibur Flow Cytometer (BD Biosciences, San Diego, CA, USA). Data were analyzed by flowjo software (v7.6; Stanford, CA, USA).

### 2.11 Senescence‐associated beta‐galactosidase (SA‐β‐gal) staining

The SA‐β‐gal staining kit (Beyotime, Shanghai, China) was used to detect the senescent cells. In brief, cells were washed with PBS for twice and were fixed for 15 min at room temperature. After washing with PBS for three times, cells were incubated in a prepared staining solution at 37°C overnight, and then were examined under a light microscope.

### 2.12 Immunofluorescence staining

Immunofluorescence staining analyses were performed as described previously [17], and were examined under an inverted fluorescent microscope (Leica, Frankfurt, Hesse‐Darmstadt, Germany). The corresponding antibodies were shown in Table S3.

### 2.13 Western blot

Western blot analyses were performed as described previously [17]. The corresponding antibodies were shown in Table S3. The membranes were detected by Odyssey infrared imaging system (LI‐COR, Lincoln, NE, USA).

### 2.14 Immunohistochemical staining (IHC)

Through immunohistochemical staining (IHC), the level of SOX6 protein was detected in tissue microarray (No. RTCEC‐201704100) purchased from Raisedragon's Co., Ltd. (Beijing, China). In brief, the level of SOX6 protein was detected by rabbit anti‐SOX6 (1:500, Abcam, UK, #ab30455), according to the protocol of MaxVision TM HRP‐Polymer anti‐Rabbit IHC Kit (Maixin, Fuzhou, China).

### 2.15 Animal experiments

The xenograft tumor experiment used for analyzing the effect of SOX6 in the tumorigenesis was performed as described previously [16, 17]. Six‐week‐old female BALB/c nude mice (CAnN.Cg‐*Foxn1* ^*nu*^/Crl) (*n* = 24) were purchased from Beijing Vital River Laboratory Animal Technology Co., Ltd. (Beijing, China).

For the effect of ABT‐263 in improving the sensitivity of SOX6‐induced senescent CC cells to cisplatin treatment, HeLa‐HA‐SOX6‐tet cells (5 × 10^6^ cells/mouse) were subcutaneously injected into the left flank of 15 six‐week‐old female BALB/c nude mice (CAnN.Cg‐*Foxn1* ^*nu*^/Crl) (Beijing Vital River Laboratory Animal Technology Co., Ltd.). After 1 week of injection, all mice were intraperitoneally injected with Dox (20 mg·kg^−1^), and then were randomly divided into three groups (*n* = 5/group). After 2 weeks of injection, all mice were still intraperitoneally injected with Dox (20 mg·kg^−1^), but were intraperitoneally injected with cisplatin (3 mg·kg^−1^, saline as solvent control) and/or orally administrated with ABT‐263 (100 mg·kg^−1^, 60% Phosal PG, 30% PEG 400 and 10% ethanol as solvent control) every other day for the next 3 weeks. After 5 weeks of injection, the mice were sacrificed under anesthesia, and the tumor blocks were collected.

The mice were kept in the specific pathogen free animal facility with 12/12 h light/dark cycle. The animal facility is regularly tested for standard pathogens. Mice were fed no more than five per cage with free access to sterile water and food. Animal experiments were approved by the Ethics Committee of Peking University (Approval No. LA2016142) and were carried out according to the guidelines established by the Institutional Animal Care and Use Committee at Peking University Health Science Center.

### 2.16 Statistical analyses

Data were shown as the mean ± standard error of mean (SEM). Group comparisons were analyzed by two‐tailed Student's *t*‐tests (two groups) or one‐way ANOVA and *post hoc* Tukey's tests (> two groups), using graphpad prism 8.0 software (GraphPad Software, San Diego, CA, USA). The *P* < 0.05 was considered statistically significant. Tumor datasets were downloaded from Gene Expression Omnibus (GEO, https://www.ncbi.nlm.nih.gov/geo) or cBioPortal database (https://www.cbioportal.org/). ALGGEN‐PROMO (https://alggen.lsi.upc.es/cgi‐bin/promo_v3/promo/promoinit.cgi?dirDB=TF_8.3) online bioinformatics tool was used for identifying the potential binding transcription factors on the promoters of the *TGFB2* and *WT1* genes.

## 3 Results

### 3.1 SOX6 induces the senescence of CC cells

**Fig. 1:**
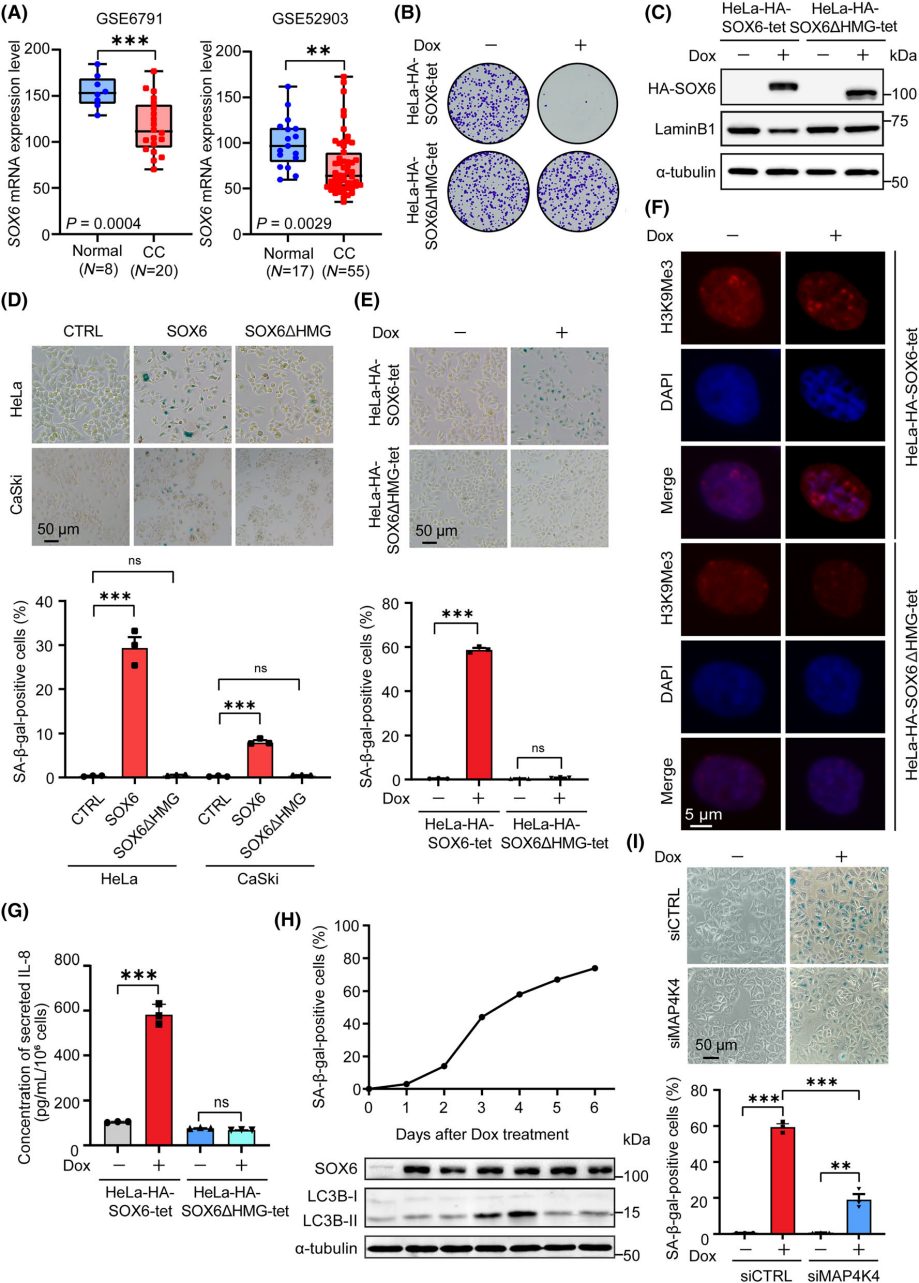
SOX6 induces the senescence of CC cells. (A) The expression levels of *SOX6* mRNA in cervical cancer (CC) tissues and normal cervical tissues were analyzed in GEO datasets. (B) HeLa‐HA‐SOX6‐tet and HeLa‐HA‐SOX6ΔHMG‐tet cells were plated into six‐well plates and were treated with Dox (2 μg·mL^−1^) or solvent control. After incubating for 2 weeks, the cell colonies were stained with 1% crystal violet and observed under natural light and (C) the levels of SOX6 and LaminB1 proteins were detected by western blot. (D) HeLa and CaSki cells were transfected with plex‐HA‐SOX6, plex‐HA‐SOX6ΔHMG, or vector control (CTRL). Four days later, the senescent cells were analyzed by SA‐β‐gal staining. (E) HeLa‐HA‐SOX6‐tet and HeLa‐HA‐SOX6ΔHMG‐tet cells were treated with Dox (2 μg·mL^−1^) or solvent control. Four days later, the senescent cells were analyzed by SA‐β‐gal staining, (F) and the SAHF formation was assessed by H3K9me3 staining. (G) HeLa‐HA‐SOX6‐tet and HeLa‐HA‐SOX6ΔHMG‐tet cells were treated with Dox (2 μg·mL^−1^) or solvent control, and the level of IL‐8 protein in the culture supernatant were detected by ELISA. (H) During the 6 days of Dox treatment, the percentage of senescent HeLa‐HA‐SOX6‐tet cells was analyzed by SA‐β‐gal staining at each day. At the same time, the levels of LC3B‐I and LC3B‐II proteins were analyzed by western blot. (I) HeLa‐HA‐SOX6‐tet cells were transfected with *MAP4K4*‐specific siRNA (siMAP4K4) or control siRNA (siCTRL), and were treated with Dox (2 μg·mL^−1^) or solvent control for 4 days. The senescent cells were analyzed by SA‐β‐gal staining. α‐tubulin was used as the internal control in western blot analysis. The percentage of SA‐β‐gal‐positive cells was analyzed at three fields. Data were shown as mean ± SEM of three independent experiments. **P < 0.01, ***P < 0.001, ns, nonsignificant, Student's *t*‐test. CC, cervical cancer; Dox, doxycycline; SAHF, senescence‐associated heterochromatin foci.

We previously found that SOX6 inhibited the proliferation of CC cells by inhibiting the transition of cell cycle from G1 to S phase [16]. The expression of the *SOX6* gene was downregulated in CC tissues, and the overall survival time of CC patients with a high SOX6 level was longer than that of patients with a low SOX6 level [27]. Based on GEO database (GSE6791 and GSE52903), we demonstrated that the level of *SOX6* mRNA in CC tissues was significantly lower than that in normal cervical tissues (Fig. 1A). In 293 CC samples from the cBioPortal database, 35.2% (102/293) had shallow deletion of the *SOX6* gene, and the levels of *SOX6* mRNA in these samples were significantly lower than those in samples with diploid *SOX6* gene (Fig. S1A). Besides, there was a positive correlation between *SOX6* mRNA level and its gene copy number (Fig. S1B), indicating that the genetic alterations might contribute to the low level of *SOX6* mRNA in CC. Furthermore, using tissue microarray, we found that the level of SOX6 protein in human CC tissues was also significantly lower than that in normal cervical tissues (Fig. S2A,B).

To further confirm the role of SOX6 in inhibiting the proliferation of CC cells, we found that doxycycline (Dox)‐mediated conditional expression of SOX6 could significantly inhibit the colony formation of HeLa‐HA‐SOX6‐tet cells, but not for SOX6 protein with HMG domain deleted (ΔHMG) in HeLa‐HA‐SOX6ΔHMG‐tet cells (Fig. 1B,C, and Fig. S3). Furthermore, we found that E6 and E7 proteins, two viral proteins expressed from the integrated HPV genome in HeLa cells, could significantly inhibit SOX6 expression (Fig. S4A–C). The level of endogenous SOX6 protein in HeLa cells with E6 or E7 expression knocked down was in a range comparable to the level of Dox‐induced SOX6 protein in HeLa‐HA‐SOX6‐tet cells. Meanwhile, the level of endogenous SOX6 protein in C‐33A cells, an HPV‐negative cervical cancer cell line with p53 mutation, was also in a range comparable to the level of Dox‐induced SOX6 protein in HeLa‐HA‐SOX6‐tet cells (Fig. S4B,C). Therefore, although Dox could induce SOX6 expression strongly, SOX6 overexpression was not robust due to the high levels of E6 and E7 proteins expressed from the integrated HPV genome in HeLa‐HA‐SOX6‐tet cells. Next, we found that overexpressing SOX6 could significantly increase the percentage of senescent HeLa and CaSki cells, but not for SOX6ΔHMG (Fig. 1D). The conditional expression of SOX6 could also significantly increase the percentage of senescent HeLa‐HA‐SOX6‐tet, CaSki‐HA‐SOX6‐tet, and SiHa‐HA‐SOX6‐tet cells (Fig. 1E and Fig. S5A,B). Meanwhile, SOX6 promoted the formation of senescence‐associated heterochromatin foci (SAHF) in the nuclei of HeLa‐HA‐SOX6‐tet cells, depending on its HMG domain (Fig. 1F). Besides, SOX6 could significantly decreased the level of LaminB1 protein, a cellular senescence‐specific protein marker [28], and could significantly increase the secreted level of IL‐8, an SASP factor, depending on its HMG domain (Fig. 1C,G).

Since we previously found that SOX6 could promote the autophagy of CC cells through the MAP4K4–ERK/AKT–mTOR pathway [17], and autophagy is one of the initial steps of some senescent cells, the effects of SOX6 in autophagy and senescence of CC cells were dynamically monitored. The results showed that the percentage of SOX6‐induced senescent HeLa‐HA‐SOX6‐tet cells gradually increased; while the level of LC3B‐II protein, an autophagy‐specific protein, gradually increased in the first 4 days of Dox treatment, but then decreased (Fig. 1H). Furthermore, the SOX6‐induced senescence of HeLa‐HA‐SOX6‐tet cells could be attenuated by knocking down the expression of endogenous MAP4K4 (Fig. 1I) and under the treatment of autophagy‐specific inhibitors Baf A1 and hydroxychloroquine (HCQ) (Fig. S5C,D).

**Fig. 2:**
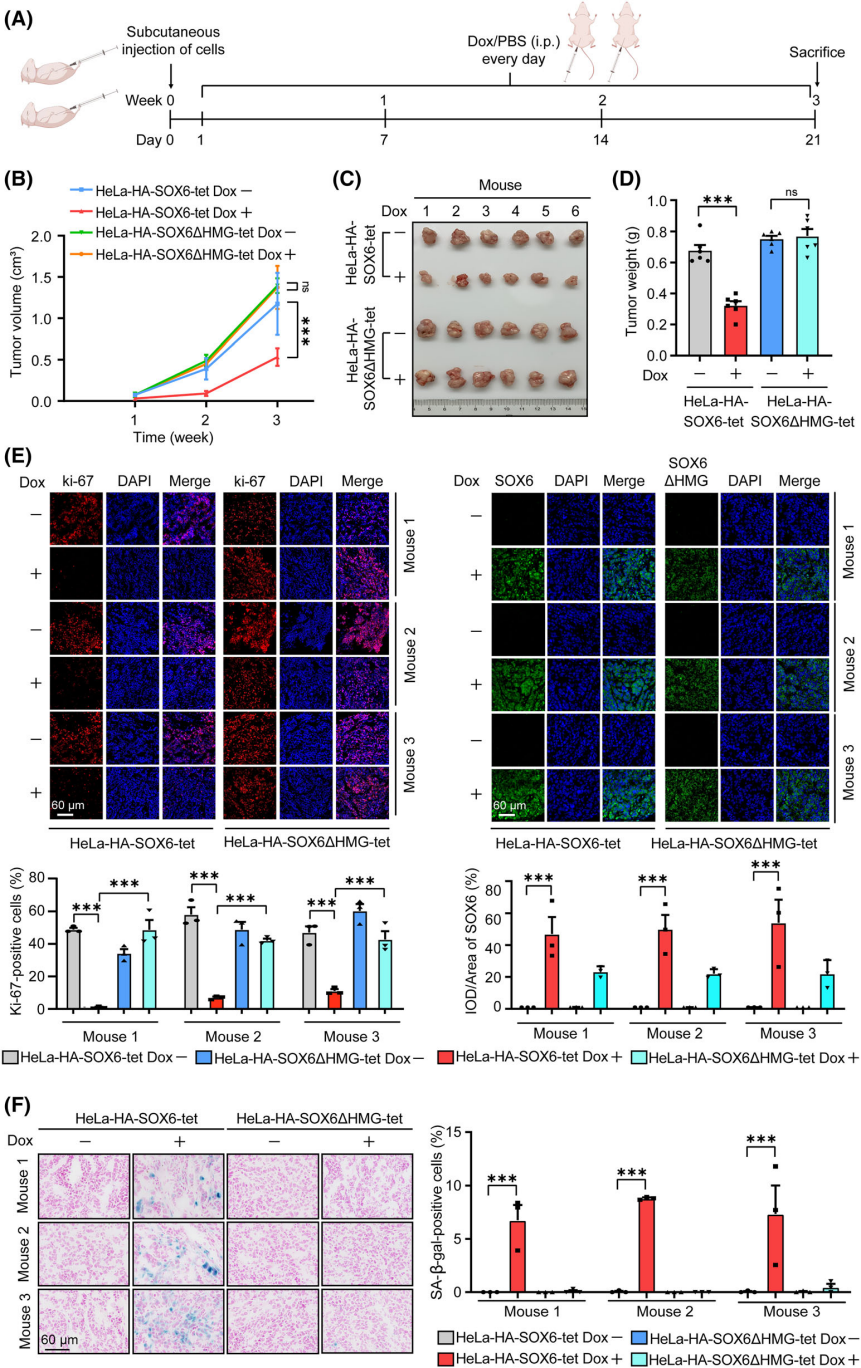
SOX6 induces the senescence of CC cells *in vivo*. (A) The procedure of xenograft tumor experiment in BALB/c nude mice. In detail, HeLa‐HA‐SOX6‐tet or HeLa‐HA‐SOX6ΔHMG cells (3 × 10^6^ cells/mice) were subcutaneously injected into the left flank of 12 nude mice. On the next day, the mice were divided into two groups (*n* = 6/group). One group of mice were intraperitoneally injected with Dox (20 mg·kg^−1^·day^−1^), and the other group of mice were intraperitoneally injected with solvent control (PBS) for 3 weeks. (B) The volumes of xenograft tumors were measured and calculated every week. (C) After 3 weeks of injection, the mice were sacrificed under anesthesia, and the tumor blocks were collected. (D) The weights of tumor blocks were measured. (E) Immunofluorescence staining of Ki‐67 (red), SOX6 or SOX6ΔHMG protein (green) in tumor tissues. The percentage of Ki‐67‐positive cells was analyzed at three fields, and the relative SOX6 or SOX6ΔHMG protein level was analyzed by calculating integrated optical density per stained area (IOD/Area). (F) The senescent cells in tumor tissues were detected by SA‐β‐gal staining assays, and the percentage of SA‐β‐gal‐positive cells was analyzed at three fields. Data were shown as mean ± SEM of three independent experiments. ***P < 0.001; ns, nonsignificant; Student's *t*‐test. CC, cervical cancer; Dox, doxycycline; i.p., intraperitoneal injection.

To further verify the role of SOX6 in inducing senescence of CC cells *in vivo*, HeLa‐HA‐SOX6‐tet and HeLa‐HA‐SOX6ΔHMG‐tet cells were subcutaneously injected into the left flank of 12 BALB/c nude mice, respectively (Fig. 2A). As shown in Fig. 2B–D, SOX6 could significantly reduce the volume, size, and weight of xenograft tumor in mice, depending on its HMG domain. The percentage of Ki‐67 positive cells in the tumors formed by the Dox‐treated HeLa‐HA‐SOX6‐tet cells was significantly lower than that in the tumors formed by the solvent control‐treated HeLa‐HA‐SOX6‐tet cells, and the percentage of senescent cells in the tumors formed by the Dox‐treated HeLa‐HA‐SOX6‐tet cells was significantly higher than that in the tumors formed by the solvent control‐treated HeLa‐HA‐SOX6‐tet cells, but there were no significant differences between the tumors formed by HeLa‐HA‐SOX6ΔHMG‐tet cells with and without Dox treatment (Fig. 2E,F).

### 3.2 TGFβ2–Smad2/3–p53–p21^WAF1/CIP1^ pathway mediates the SOX6‐induced cellular senescence

**Fig. 3:**
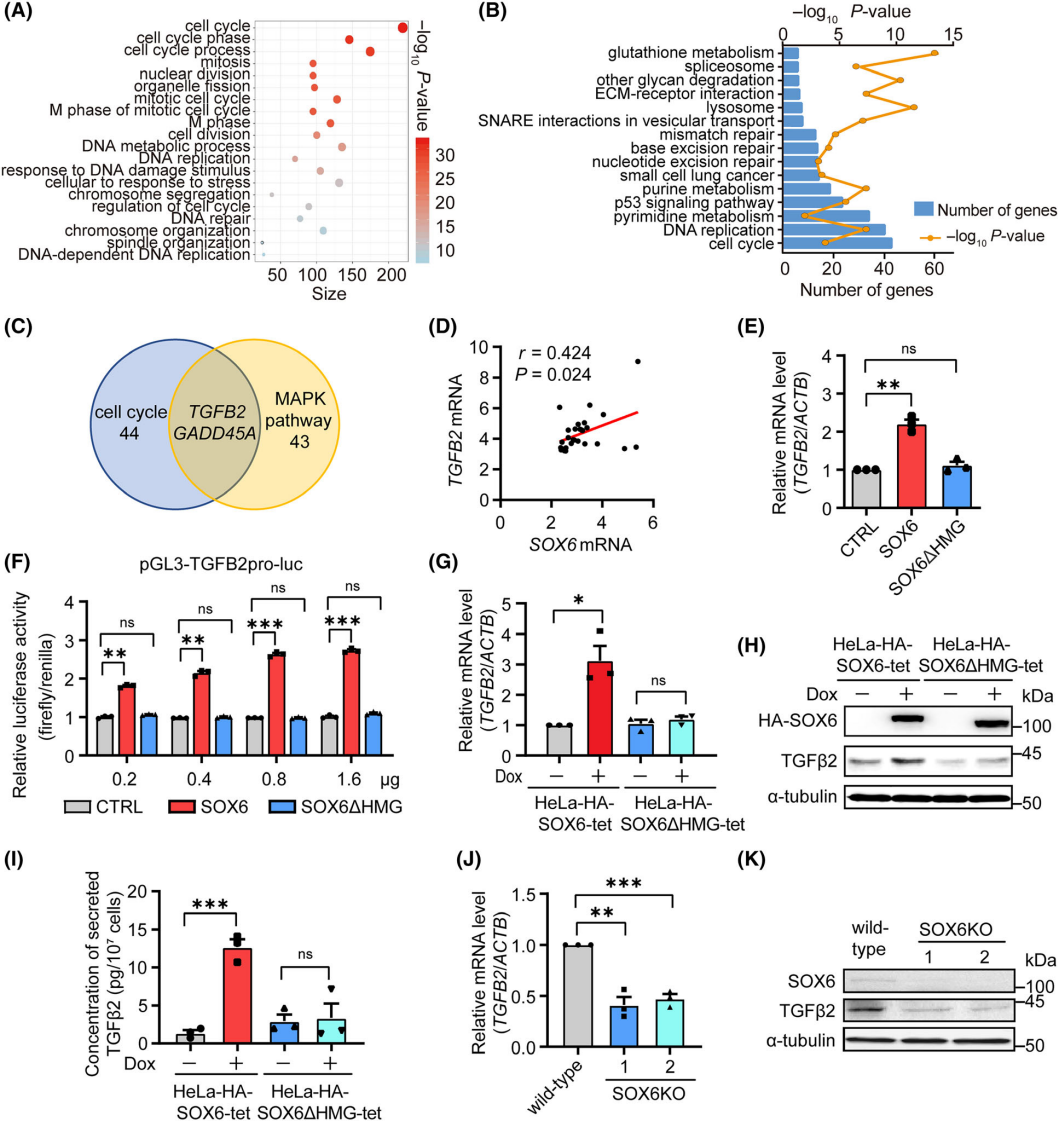
*TGFB2* gene is a potential target gene of SOX6. (A) The diagram of top 20 GO enrichment biological processes in microarray analysis. (B) The diagram of top 15 KEGG enrichment pathways in microarray analysis. (C) The Venn diagram of DEGs enriched in cell cycle and MAPK pathways. (D) The correlation between the levels of *SOX6* mRNA and *TGFB2* genes in 28 CC tissues from the GEO database was analyzed by Spearman correlation analysis. (E) HeLa cells were transfected with plex‐HA‐SOX6, plex‐HA‐SOX6ΔHMG, or vector control (CTRL), and the level of *TGFB2* mRNA was detected by RT‐qPCR. (F) The pGL3‐TGFB2pro‐luc, PRL‐TK (Renilla), and 0.2, 0.4, 0.8, or 1.6 μg of plex‐HA‐SOX6, plex‐HA‐SOX6ΔHMG, or vector control (CTRL) were cotransfected into HeLa cells, and the transcriptional activity of *TGFB2* gene promoter was analyzed by dual‐luciferase assay. (G) HeLa‐HA‐SOX6‐tet and HeLa‐HA‐SOX6ΔHMG‐tet cells were treated with Dox (2 μg·mL^−1^) or solvent control, and the levels of *TGFB2* mRNA, (H) intracellular TGFβ2 protein, (I) and TGFβ2 protein in the culture supernatant were detected by RT‐qPCR, western blot, and ELISA, respectively. (J) The levels of *TGFB2* mRNA and (K) TGFβ2 protein in HeLa cells with or without *SOX6* gene knocked out were detected by RT‐qPCR and western blot, respectively. *ACTB* mRNA and α‐tubulin was used as the internal control for RT‐qPCR and western blot, respectively. Data were shown as mean ± SEM of three independent experiments. *P < 0.05; **P < 0.01; ***P < 0.001; ns, nonsignificant; Student's *t*‐test. CC, cervical cancer; DEGs, differentially expressed genes; Dox, doxycycline; GO, gene ontology.

Based on our previous microarray analysis (GSE223837) on the genome‐wide expression profile of HeLa cells overexpressing SOX6 [17], a total of 4488 differentially expressed genes (DEGs) (< 0.5‐ or > 2.0‐fold, *P* < 0.05) were identified and subsequently analyzed by gene ontology (GO) and KEGG pathway enrichment analyses to explore the underlying mechanism of SOX6‐induced cellular senescence. In GO enrichment analysis, cell cycle and its related biological processes were the most significant among the top 20 enriched biological processes (Fig. 3A). In KEGG pathway enrichment analysis, the number of genes in the cell cycle pathway was the most among the top 15 pathways (Fig. 3B). Since the MAP4K4 pathway also mediated SOX6‐induced cellular senescence (Fig. 1I), we merged the DEGs enriched in the cell cycle and MAPK pathways. The result showed that the *TGFB2* and *GADD45A* genes were the two overlapping DEGs (Fig. 3C). It is well known that the *GADD45A* gene is a downstream gene of cell cycle pathway [29, 30]. Therefore, the *TGFB2* gene was selected as a candidate target gene mediating the SOX6‐induced senescence of CC cells.

Consistent with the result of microarray analysis, a positive correlation (*r* = 0.424, *P* = 0.024) between *SOX6* and *TGFB2* mRNA levels in CC tissues was found by analyzing GEO database (GSE63514) (Fig. 3D), and SOX6 could significantly upregulate the level of *TGFB2* mRNA in HeLa cells, depending on its HMG domain (Fig. 3E). Furthermore, SOX6 could significantly enhance the transcriptional activity of the *TGFB2* gene promoter in a dose‐dependent manner, suggesting that SOX6 promoted the expression of the *TGFB2* gene at the transcriptional level (Fig. 3F). Consistently, the Dox‐induced conditional expression of SOX6 could also promote the expression of the *TGFB2* gene at both the mRNA and protein levels in HeLa‐HA‐SOX6‐tet cells, depending on its HMG domain (Fig. 3G,H, and Fig. S6A). SOX6 could also increase the level of TGFβ2 protein, a secretory protein, in the culture supernatant of HeLa cells (Fig. 3I). However, the expression of the *TGFB2* gene was suppressed in HeLa cells when the *SOX6* gene was knocked out by CRISPR/Cas9 technique (Fig. 3J,K).

**Fig. 4:**
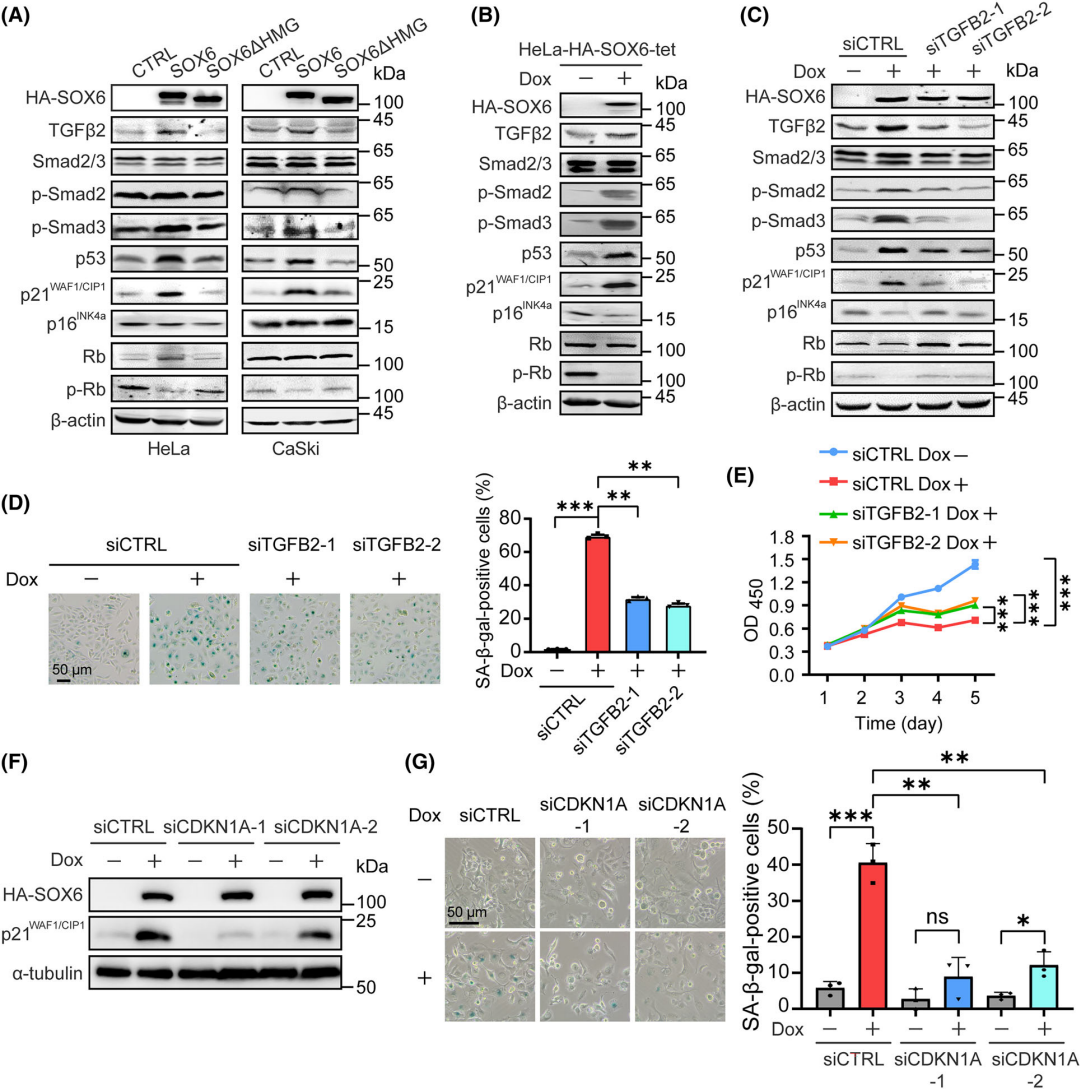
TGFβ2–Smad–p53–p21^WAF1/CIP1^ pathway mediates the SOX6‐induced senescence of CC cells. (A) HeLa and CaSki cells were transfected with plex‐HA‐SOX6, plex‐HA‐SOX6ΔHMG, or vector control (CTRL), (B) HeLa‐HA‐SOX6 cells were treated with Dox (2 μg·mL^−1^) or solvent control, (C) and HeLa‐HA‐SOX6‐tet cells were transfected with *TGFB2*‐specific siRNA‐1 (siTGFB2‐1), *TGFB2*‐specific siRNA‐2 (siTGFB2‐2), or control siRNA (siCTRL). The levels of TGFβ2–Smad signal pathway‐related proteins and senescence‐related proteins were analyzed by western blot. (D) HeLa‐HA‐SOX6‐tet cells transfected with siTGFB2‐1, siTGFB2‐2, or siCTRL were treated with Dox (2 μg·mL^−1^) or solvent control for 4 days, and the senescent cells were analyzed by SA‐β‐gal staining. (E) The cell viability was analyzed by CCK‐8 assays. (F) HeLa‐HA‐SOX6‐tet cells were transfected with *CDKN1A*‐specific siRNA‐1 (si‐CDKN1A‐1), *CDKN1A*‐specific siRNA‐2 (si‐CDKN1A‐2), or control siRNA (siCTRL) and were treated with Dox (2 μg·mL^−1^) or solvent control for 4 days. The protein levels of HA‐SOX6 and p21^WAF1/CIP1^ were measured by western blot, (G) and the senescent cells were analyzed by SA‐β‐gal staining. The percentage of SA‐β‐gal‐positive cells was analyzed at three fields. *ACTB* mRNA and β‐Actin or α‐tubulin protein was used as the internal controls for RT‐qPCR and western blot, respectively. Data were shown as mean ± SEM of three independent experiments. *P < 0.05; **P < 0.01; ***P < 0.001; ns, nonsignificant; Student's *t*‐test. CC, cervical cancer; Dox, doxycycline.

Next, we found that SOX6 could activate the TGFβ2–Smad2/3 pathway, increase the levels of senescence‐related proteins including p53 and p21^WAF1/CIP1^ proteins, and reduce the level of the phosphorylated Rb protein depending on its HMG domain, but did not obviously affect the level of p16^INK4a^ protein in HeLa and CaSki cells (Fig. 4A, and Fig. S6B–G). The similar phenomenon was also found in the Dox‐treated HeLa‐HA‐SOX6‐tet cells (Fig. 4B). Moreover, the TGFβ2‐Smad pathway could also directly increase the levels of senescence‐related proteins in HeLa cells (Fig. S7A). Such a phenomenon was attenuated when the expression of endogenous TGFβ2 was knocked down by *TGFB2*‐specific small interfering RNAs (siTGFB2‐1 and siTGFB2‐2) in the Dox‐treated HeLa‐HA‐SOX6‐tet cells (Fig. 4C). Meanwhile, the percentage of SOX6‐induced senescent cells, the SOX6‐mediated inhibition of cell proliferation, and cell cycle arrest were also significantly attenuated by knocking down the expression of endogenous TGFβ2 (Fig. 4D,E and Fig. S7B,C). The percentage of SOX6‐induced senescent cells was also significantly attenuated by knocking down the expression of endogenous p21^WAF1/CIP1^ (Fig. 4F,G). Moreover, the increase on the levels of p53 and p21^WAF1/CIP1^ proteins in the Dox‐treated HeLa‐HA‐SOX6‐tet cells could be suppressed by treating with ITD‐1, an inhibitor of Smad2/3 phosphorylation (Fig. S7D). These results suggested that the TGFβ2–Smad2/3–p53–p21^WAF1/CIP1^‐Rb pathway rather than the p16^INK4a^‐Rb pathway could mediate the SOX6‐induced cellular senescence.

### 3.3 MAP4K4 (ERK/JNK/p38)–ATF2–TGFβ2 pathway mediates the SOX6‐induced cellular senescence

HMG domain, as a DNA‐binding domain, mediates the transcriptional regulation ability of SOX6 via binding to the conservative sequence (A/T)(A/T)CAA(A/T)G of the target gene promoter [16, 17, 22]. To explore whether SOX6 promoted the expression of the *TGFB2* gene through binding of its HMG domain to the *TGFB2* gene promoter, the conservative binding sequence of SOX6 was searched at the *TGFB2* gene promoter. As shown in Fig. S8A, a potential SOX6‐binding site (**AACAAAG**TACTTAACTT**CTTTGTA**) was found at 1234–1257 bp upstream of the *TGFB2* gene transcription start site (TSS). Furthermore, chromatin immunoprecipitation combined with PCR (ChIP‐PCR) assays confirmed that SOX6 could bind to this potential binding site, depending on its HMG domain (Fig. S8B). However, mutating the SOX6‐binding site could not prevent SOX6 from enhancing the transcriptional activity of the *TGFB2* gene promoter (Fig. S8C).

**Fig. 5:**
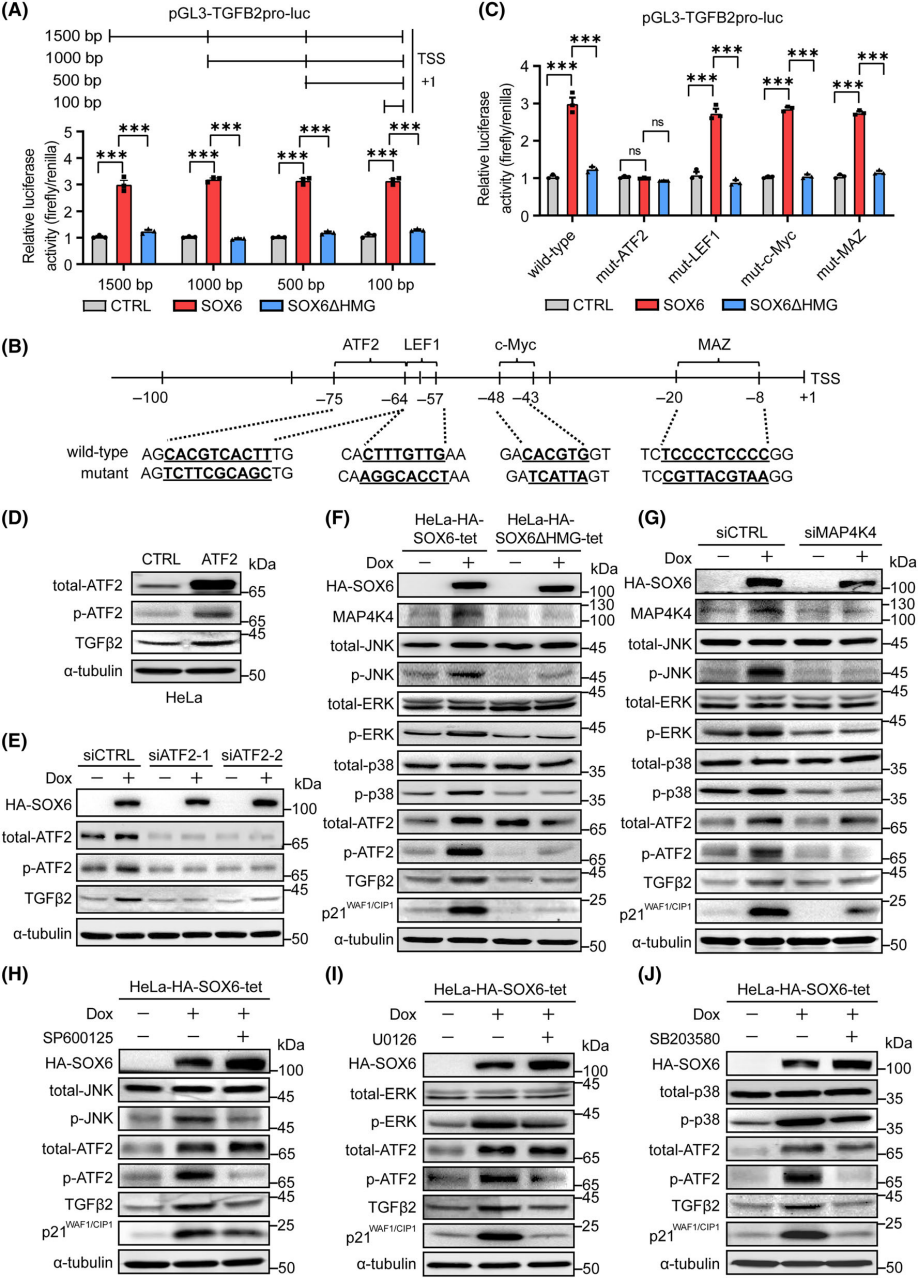
MAP4K4 (JNK/ERK/p38)–ATF2 pathway mediates SOX6 to promote the expression of *TGFB2* gene. (A) Luciferase reporter plasmid fused to 1500, 1000, 500, or 100 bp sequence upstream of the *TGFB2* gene TSS (pGL3‐TGFB2pro‐luc) was cotransfected with PRL‐TK (Renilla) and plex‐HA‐SOX6, plex‐HA‐SOX6ΔHMG or vector control (CTRL) in HeLa cells, respectively. The transcriptional activity of *TGFB2* gene promoter was detected by dual‐luciferase assay. (B) The wild‐type and mutant sequences of ATF2, LEF1, c‐Myc and MAZ binging sites at 100 bp upstream of the *TGFB2* gene TSS. (C) pGL3‐TGFB2pro‐luc (wild‐type), pGL3‐TGFB2pro‐mutant (ATF2)‐luc (mut‐ATF2), pGL3‐TGFB2pro‐mutant(LEF1)‐luc (mut‐LEF1), pGL3‐TGFB2pro‐mutant(c‐Myc)‐luc (mut‐c‐Myc), or pGL3‐TGFB2pro‐mutant(MAZ)‐luc (mut‐MAZ) plasmid was cotransfected with PRL‐TK and plex‐HA‐SOX6, plex‐HA‐SOX6ΔHMG, or vector control (CTRL) in HeLa cells, respectively. The transcriptional activity of *TGFB2* gene promoter was detected by dual‐luciferase assay. (D) The pCDH‐ATF2 plasmid or vector control (CTRL) was transfected into HeLa cells. The levels of total‐ATF2, p‐ATF2, and TGFβ2 proteins were detected by western blot. (E) The *ATF2*‐specific siRNA (siATF2‐1 or siATF2‐2) or control siRNA (siCTRL) was transfected into HeLa‐HA‐SOX6‐tet cells and were treated with Dox (2 μg·mL^−1^) or solvent control for 4 days. The levels of total‐ATF2, p‐ATF2, and TGFβ2 proteins were detected by western blot. (F) HeLa‐HA‐SOX6‐tet and HeLa‐HA‐SOX6ΔHMG‐tet cells were treated with Dox (2 μg·mL^−1^) or solvent control. (G) HeLa‐HA‐SOX6‐tet cells were transfected with siCTRL or siMAP4K4 and were treated with Dox (2 μg·mL^−1^) or solvent control. The levels of key proteins in the MAP4K4 (JNK/ERK/p38)–ATF2–TGFβ2 pathway were detected by western blot. (H) HeLa‐HA‐SOX6‐tet cells were treated with Dox (2 μg·mL^−1^) or solvent control and SP600125 (5 μM), (I) U0126 (5 μM), (J) or SB203580 (5 μM). The corresponding protein levels were detected by western blot. Data were shown as mean ± SEM of three independent experiments. ***P < 0.001; ns, nonsignificant; Student's *t*‐test. Dox, doxycycline; TSS, transcription start site.

To explore the potential transcription factors (TFs) mediating SOX6 to transcriptionally promote the expression of the *TGFB2* gene, we constructed four luciferase reporter plasmids with different lengths of sequences located at 0–1500, 0–1000, 0–500, and 0–100 bp upstream of the *TGFB2* gene TSS, respectively. As shown in Fig. 5A, SOX6 could enhance the transcriptional activity of four *TGFB2* gene promoters with different lengths to the same extent, suggesting that the binding sites of potential TFs might be located at 0–100 bp upstream of the *TGFB2* gene TSS. Using ALGGEN‐PROMO database, the potential binding sites of four TFs were found at 0–100 bp upstream of the *TGFB2* gene TSS, including lymphoid enhancer‐binding factor 1 (LEF1), c‐Myc, MYC‐associated zinc finger protein (MAZ), and activating transcription factor‐2 (ATF2) (Fig. 5B). It has been reported that LEF‐1 is an important TF in the Wnt/β–catenin signal pathway [31], c‐Myc is an important TF in the processes of cell cycle, apoptosis, and senescence [32], MAZ regulates the transcriptional initiation and termination of the *MYC* gene [33], and ATF2 participates in tumor formation and progression [34, 35]. To further explore which TF mediated SOX6 to transcriptionally promote the expression of the *TGFB2* gene, luciferase reporter plasmids with mutated binding sites of four TFs were constructed, respectively (Fig. 5B). The results showed that the SOX6‐enhanced transcriptional activity of the *TGFB2* gene promoter was only attenuated when ATF2‐binding site was mutated (Fig. 5C). Furthermore, the level of TGFβ2 protein not only increased by overexpressing ATF2 (Fig. 5D and Fig. S9A) but also decreased by knocking down the expression of the endogenous *ATF2* gene (Fig. 5E).

ATF2, as a downstream effector of the MAPK pathway, can be phosphorylated by ERK/JNK/p38 signal cascade, and can transcriptionally regulate target gene expression through binding of the phosphorylated ATF2 protein to the target gene promoter [36, 37, 38]. As shown in Fig. 5F and Fig. S9B–D, SOX6 could activate MAP4K4 (ERK/JNK/p38) signal cascade and promote the phosphorylation of ATF2 protein in HeLa‐HA‐SOX6‐tet cells, depending on its HMG domain. Consistently, SOX6 could also promote MAP4K4 expression and ATF2 phosphorylation in CaSki‐HA‐SOX6‐tet and SiHa‐HA‐SOX6‐tet cells (Fig. S10A,B). However, the SOX6‐mediated activation of the MAP4K4 (ERK/JNK/p38) pathway and phosphorylation of ATF2 protein could be attenuated by knocking down the expression of the endogenous *MAP4K4* gene (Fig. 5G). The SOX6‐mediated phosphorylation of ATF2 protein could also be attenuated by inhibiting the activities of JNK, ERK, and p38 kinases with the corresponding inhibitor SP600125 (Fig. 5H), U0126 (Fig. 5I), and SB203580 (Fig. 5J), respectively. The percentage of senescent cells induced by SOX6 also significantly decreased when the activities of MAP4K4, JNK, ERK, and p38 kinases were inhibited by the corresponding inhibitors (Fig. S10C,D), and inhibiting MAP4K4 could significantly decrease cell viability and promote cell apoptosis of the Dox‐treated HeLa‐HA‐SOX6‐tet cells (Fig. S11A–C). These results suggested that the MAP4K4 (ERK/JNK/p38)–ATF2–TGFβ2 pathway could mediate the SOX6‐induced senescence of CC cells, and the MAP4K4 inhibitors might be the potential senolytic agents.

### 3.4 WT1–ATF2–TGFβ2 pathway mediates the SOX6‐induced cellular senescence

As shown in Fig. 5F, SOX6 could not only promote the phosphorylation of ATF2 protein but also increase the level of total ATF2 protein. Although the MAP4K4 (ERK/JNK/p38) pathway mediated SOX6 to promote the phosphorylation of ATF2 protein, it had no effect on the level of total ATF2 protein (Fig. 5G–J). Therefore, there might be another pathway mediating SOX6 to increase the level of total ATF2 protein.

**Fig. 6:**
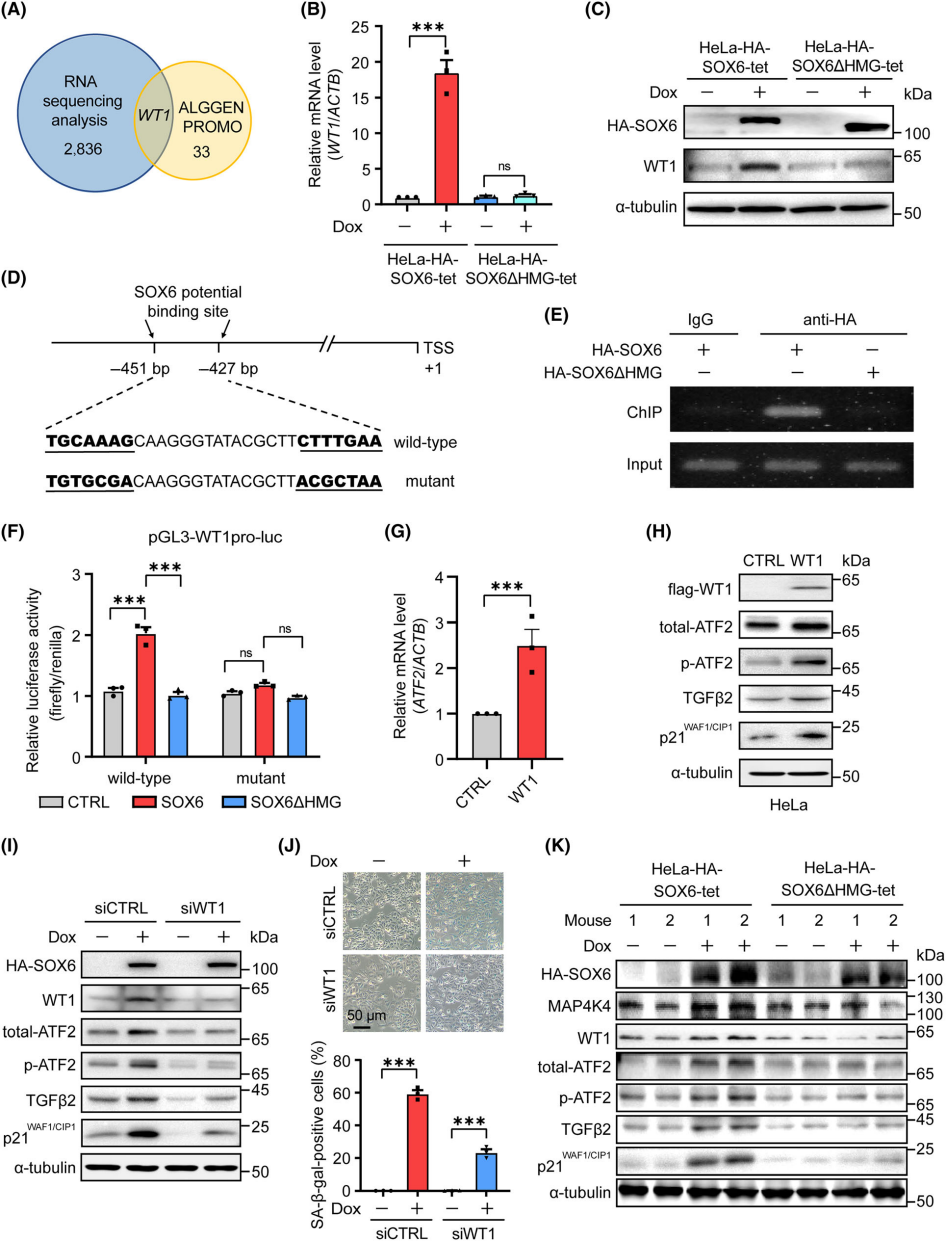
WT1–ATF2–TGFβ2 pathway mediates the SOX6‐induced cellular senescence. (A) Merging 33 TFs predicted by ALGGEN‐PROMO database that potentially bind to the *ATF2* gene promoter and 2836 DEGs identified by RNA‐sequencing analysis of HeLa‐HA‐SOX6‐tet cells treated with Dox (2 μg·mL^−1^) or solvent control. (B) HeLa‐HA‐SOX6‐tet and HeLa‐HA‐SOX6ΔHMG‐tet cells were treated with Dox (2 μg·mL^−1^) or solvent control. The level of *WT1* mRNA was analyzed by RT‐qPCR, (C) and the level of WT1 protein was analyzed by western blot. (D) The wild‐type and mutant sequences of the potential SOX6‐binding site at 427–457 bp upstream of the *WT1* gene TSS. (E) The plex‐HA‐SOX6 or plex‐HA‐SOX6ΔHMG plasmid was transfected into HeLa cells. HA‐labeled SOX6 or SOX6ΔHMG protein were immunoprecipitated by anti‐HA tag antibody, and then the potential binding region of SOX6 was amplified by PCR. The PCR products were detected by 1.5% agarose gel electrophoresis. (F) The pGL3‐WT1pro‐luc (wild‐type) or pGL3‐WT1pro‐mutant (SOX6)‐luc (mutant), and plex‐HA‐SOX6, plex‐HA‐SOX6ΔHMG or vector control (CTRL) plasmids were cotransfected into HeLa cells. The transcriptional activity of *WT1* gene promoter was detected by dual‐luciferase assay. (G) The pCDH‐flag‐WT1 (WT1) or vector control (CTRL) plasmid was transfected into HeLa cells. The level of *ATF2* mRNA was detected by RT‐qPCR, (H) and the levels of WT1, total‐ATF2, p‐ATF2, TGFβ2, and p21^WAF1/CIP1^ proteins were detected by western blot. (I) HeLa‐HA‐SOX6‐tet cells were transfected with siRNA control (siCTRL) or *WT1* mRNA‐specific siRNA (siWT1), and then were treated with Dox (2 μg·mL^−1^) or solvent control for 4 days. The corresponding proteins were detected by western blot. (J) The senescent cells were detected by SA‐β‐gal staining assays, and the percentage of SA‐β‐gal‐positive cells was analyzed at three fields. (K) The levels of key proteins in the MAP4K4/WT1–ATF2–TGFβ2 pathways in xenograft tumor tissues were detected by western blot. Data were shown as mean ± SEM of three independent experiments. ***P < 0.001; ns, nonsignificant; Student's *t*‐test. DEGs, differentially expressed genes; Dox, doxycycline; TF, transcription factor; TSS, transcription start site.

To explore this pathway, we first found that SOX6 could significantly increase the level of *ATF2* mRNA and enhance the transcriptional activity of the *ATF2* gene promoter, depending on its HMG domain (Fig. S12A,B). However, there was no SOX6‐binding site at the *ATF2* gene promoter. To explore the potential TFs mediating SOX6 to promote the expression of the *ATF2* gene, we merged 33 TFs predicted by ALGGEN‐PROMO database that potentially bind to the *ATF2* gene promoter and 2836 SOX6‐induced DEGs identified by RNA‐sequencing analysis (HRA001566). As shown in Fig. 6A, only WT1 transcription factor (*WT1*) gene was screened as a potential TF‐mediating SOX6 to promote the expression of the *ATF2* gene. SOX6 could significantly increase both the mRNA and protein levels of the *WT1* gene, depending on its HMG domain (Fig. 6B,C, and Fig. S12C). Through analyzing the sequence of the *WT1* gene promoter, the potential SOX6‐binding site was found at 427–451 bp upstream of the *WT1* gene TSS (Fig. 6D). ChIP‐PCR confirmed that SOX6 could bind to the *WT1* gene promoter, depending on its HMG domain (Fig. 6E). Further, dual‐luciferase assays showed that SOX6 could significantly enhance the transcriptional activity of the *WT1* gene promoter, but this effect disappeared when the SOX6‐binding site was mutated (Fig. 6D,F). Next, we confirmed that WT1 could bind to the *ATF2* gene promoter and significantly increase the mRNA and protein levels of the *ATF2* gene (Fig. 6G,H, and Fig. S12D–G). However, the SOX6‐induced increases on the levels of total ATF2 protein and its downstream TGFβ2 protein were obviously attenuated by knocking down the expression of the endogenous *WT1* gene (Fig. 6I). Meanwhile, the percentage of SOX6‐induced senescent cells significantly decreased when the expression of the endogenous *WT1* gene was knocked down (Fig. 6J). These results suggested that the WT1–ATF2–TGFβ2 pathway could also mediate the SOX6‐induced senescence of CC cells.

To further verify the mechanism on the SOX6‐induced senescence of CC cells, we detected the effects of SOX6 in the MAP4K4/WT1–ATF2–TGFβ2 pathways in above xenograft tumor tissues. The results showed that SOX6 rather than SOX6ΔHMG could also increase the levels of MAP4K4, WT1, total ATF2, phosphorylated ATF2, TGFβ2, and p21^WAF1/CIP1^ proteins *in vivo* (Fig. 6K). These results further demonstrated that SOX6 could induce the senescence of CC cells via the MAP4K4/WT1–ATF2–TGFβ2 pathways.

### 3.5 Senolytics treatment can overcome the cisplatin resistance mediated by SOX6‐induced cellular senescence

**Fig. 7:**
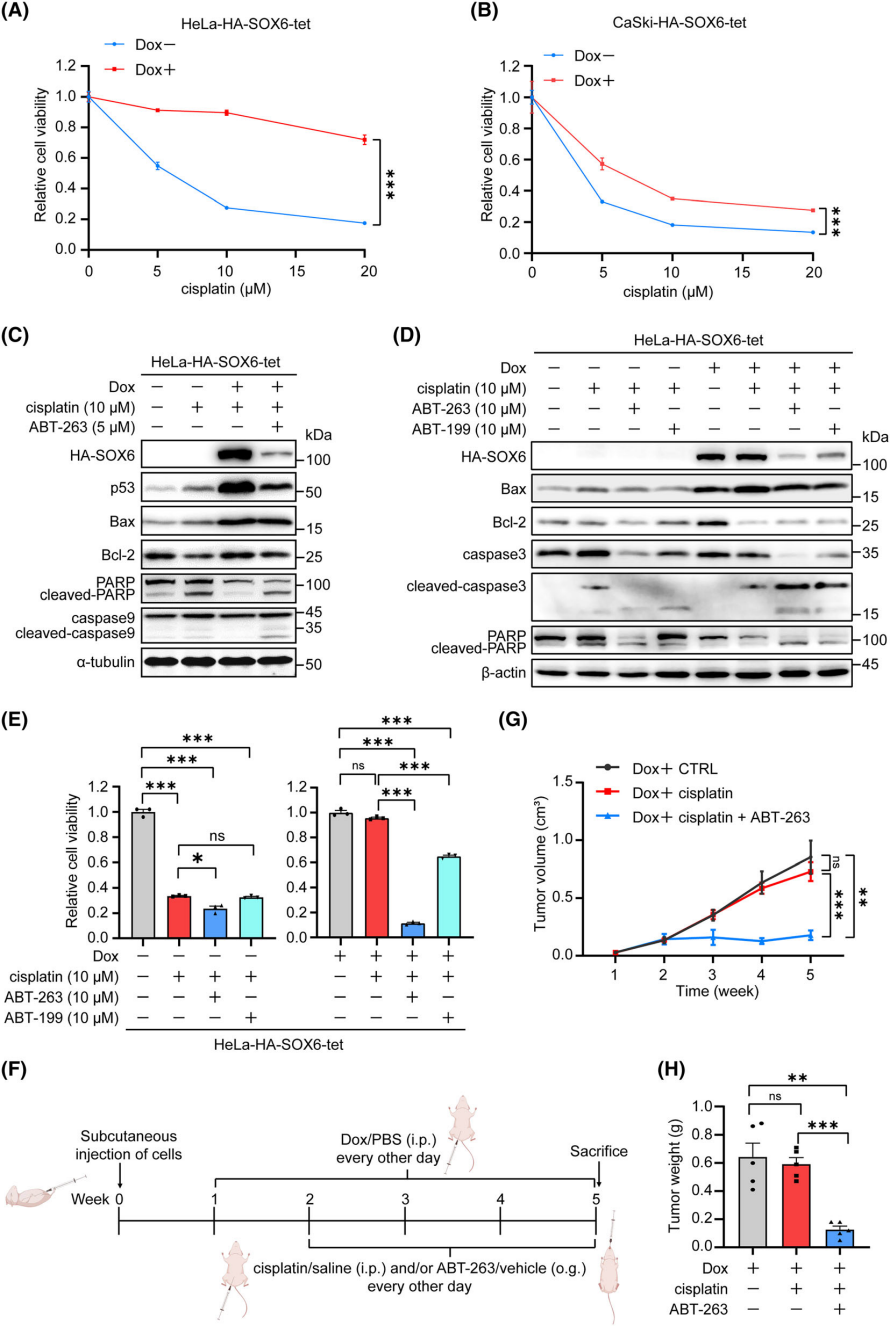
ABT‐263 and ABT‐199 overcome the SOX6‐mediated cisplatin resistance. (A) HeLa‐HA‐SOX6‐tet and (B) CaSki‐HA‐SOX6‐tet cells were treated with gradient concentration of cisplatin, and the cell viability was assessed by CCK‐8 assays. (C) HeLa‐HA‐SOX6‐tet cells were pretreated with Dox (2 μg·mL^−1^) or solvent control, and then were treated with cisplatin (10 μM) and ABT‐263 (5 μM) or solvent control. The levels of apoptosis‐related proteins were detected by western blot. α‐tubulin was used as the internal control. (D) HeLa‐HA‐SOX6‐tet cells were pretreated with Dox (2 μg·mL^−1^) or solvent control, and then were treated with cisplatin (10 μM) and ABT‐263 (10 μM), ABT‐199 (10 μM), or solvent control. The levels of apoptosis‐related proteins were detected by western blot, β‐Actin was used as the internal control, (E) and the cell viability was assessed by CCK‐8 assays. (F) HeLa‐HA‐SOX6‐tet cells were subcutaneously injected into the left flank of 15 BALB/c nude mice. After 1 week of injection, the mice were intraperitoneally injected with Dox (20 mg·kg^−1^) for 1 week, and then the mice were divided into three groups (*n* = 5/group). The mice of group I were intraperitoneally injected with Dox and the solvent control of cisplatin, and were orally fed with the solvent control of ABT‐263 every other day; The mice of group II were intraperitoneally injected with Dox and cisplatin (3 mg·kg^−1^), and were orally fed with the solvent control of ABT‐263 every other day; The mice of group III were intraperitoneally injected with Dox and cisplatin (3 mg·kg^−1^), and were orally fed with ABT‐263 (100 mg·kg^−1^) every other day. After 3 weeks of the combined treatment with cisplatin and ABT‐263, the mice were sacrificed under anesthesia. (G) The volumes of xenograft tumors were measured every week. (H) The weights of tumor blocks were measured. Data were shown as mean ± SEM of three independent experiments. **P < 0.01; ***P < 0.001; ns, nonsignificant; Student's *t*‐test. Dox, doxycycline; i.p., intraperitoneal injection; o.g., oral gavage.

Our previous study found that the levels of endogenous SOX6 and its downstream MAP4K4 proteins in CC tissues of patients who were resistant to cisplatin treatment were significantly higher than those in patients who were sensitive to cisplatin treatment [17]. Consistently, SOX6‐induced cellular senescence could significantly reduce the sensitivity of cisplatin treatment in HeLa‐HA‐SOX6‐tet and CaSki‐HA‐SOX6‐tet cells (Fig. 7A,B). Since senolytics could selectively eliminate the senescent cancer cells [39], we further explored whether senolytics treatment could overcome the SOX6‐mediated cisplatin resistance. As shown in Fig. 7C, ABT‐263 could significantly increase the levels of apoptosis‐related proteins in the SOX6‐mediated cisplatin‐resistant HeLa‐HA‐SOX6‐tet cells, suggesting that ABT‐263 could promote apoptosis of the SOX6‐induced senescent cancer cells. In addition, the level of SOX6 protein significantly decreased under ABT‐263 treatment, indicating that ABT‐263 could efficiently eliminate the SOX6‐induced senescent cancer cells (Fig. 7C). The same phenomenon was also found for ABT‐199 treatment but was weak in those cells without Dox treatment, suggesting that the two senolytics selectively eliminated the SOX6‐induced senescent cancer cells (Fig. 7D). Consistently, both ABT‐263 and ABT‐199 could specifically decrease the cell viability of the SOX6‐mediated cisplatin‐resistant HeLa‐HA‐SOX6‐tet cells (Fig. 7E). The similar phenomenon was also found in CaSki‐HA‐SOX6‐tet cells (Fig. S13A,B). We previously found that cisplatin could promote the expression of endogenous SOX6, which could reduce the sensitivity of CC cells to cisplatin treatment in turn [17]. Since ABT‐263 and ABT‐199 could also decrease the level of cisplatin‐upregulated endogenous SOX6 protein, the two senolytics might also eliminate the cells with a high level of endogenous SOX6 protein induced by cisplatin (Fig. S13C).

The effect of ABT‐263 in overcoming the SOX6‐mediated cisplatin resistance was also explored *in vivo*. As shown in Fig. 7F, HeLa‐HA‐SOX6‐tet cells were subcutaneously injected into the left flank of 15 BALB/c nude mice, and the mice were sacrificed after 3 weeks of the combined treatment with cisplatin and ABT‐263. The results revealed that SOX6 could also induce cisplatin resistance of xenograft tumor, but the combined treatment with ABT‐263 could significantly reduce the volumes, sizes, and weights of the cisplatin‐resistant xenograft tumor in mice (Fig. 7G,H and Fig. S13D). These results demonstrated that senolytics could overcome the cisplatin resistance mediated by SOX6‐induced cellular senescence.

## 4 Discussion

In this study, we first found that SOX6 could inhibit cell proliferation by inducing senescence of CC cells *in vitro* and *in vivo*. Recent evidences show that autophagy can activate or inhibit cellular senescence, depending on the type of autophagy, cell type, or the type of inducer in triggering cellular senescence [40, 41, 42]. We previously found that SOX6 could induce autophagy of CC cells by promoting the expression of the *MAP4K4* gene, a direct target gene of SOX6 [17], which made us wonder the relationship between the SOX6‐induced autophagy and senescence of CC cells. Here, we found that MAP4K4 could also mediate the SOX6‐induced senescence following by autophagy of CC cells. It has been reported that MAP4K4 not only promotes tumorigenesis and cancer metastasis [43, 44] but also mediates the Ras‐induced cellular senescence [45]. Therefore, MAP4K4 plays an important role in maintaining cell survival after cell proliferation is inhibited, which ultimately leads to autophagy and subsequent senescence of CC cells.

Based on microarray analysis, we found that SOX6 could induce the senescence of CC cells through upregulating the expression of the *TGFB2* gene and subsequently activating the TGFβ2–Smad2/3 pathway. It has been reported that TGFβ family is involved in the control of cell proliferation, including cellular senescence, and the TGFβ–Smad3 pathway promotes cellular senescence by inhibiting the expression of the human telomerase reverse transcriptase (*hTERT*) gene and subsequently inhibiting the activity of telomerase [46, 47, 48]. Furthermore, we found that ATF2 mediated SOX6 to promote the expression of the *TGFB2* gene by binding to its promoter, and SOX6 could not only promote the phosphorylation of ATF2 protein by activating the MAP4K4 (JNK/ERK/p38) pathway but also increase the level of ATF2 protein by promoting the expression of the *WT1* gene, another direct target gene of SOX6. Therefore, the MAP4K4/WT1–ATF2–TGFβ2 pathways contribute to the SOX6‐induced senescence of CC cells. As we know, cellular senescence is mainly mediated by the p53–p21^WAF1/CIP1^–Rb and p16^INK4a^–Rb pathways [10]. Here, we found that SOX6 could induce the senescence of CC cells via the TGFβ2–Smad2/3–p53–p21^WAF1/CIP1^‐Rb pathway rather than the p16^INK4a^–Rb pathway.

Since the senescent tumor cells have a risk of promoting tumor recurrence, it is better to eliminate the senescent tumor cells in time [49]. ABT‐263 and ABT‐199 are the two classic senolytics, which can selectively eliminate the senescent cells by blocking the binding of Bcl‐2 and Bax proteins [50, 51]. In this study, we found that both ABT‐263 and ABT‐199 could efficiently induce apoptosis of the SOX6‐induced senescent CC cells. At present, cisplatin is still the first‐line chemotherapy drug in the treatment of CC [7]. We previously found that SOX6 was highly expressed in the CC tissues of cisplatin‐resistant patients and mediated the cisplatin resistance of CC cells. Meanwhile, cisplatin treatment could also increase the level of endogenous SOX6 protein, which might in turn contribute to the cisplatin resistance [17]. Here, we found that the SOX6‐induced senescent CC cells were resistant to cisplatin treatment. Both ABT‐263 and ABT‐199 could eliminate the SOX6‐induced senescent CC cells, thus senolytics could be used to increase the chemotherapy sensitivity of cisplatin‐resistant CC cells.

## 5 Conclusions

In summary, this study found that MAP4K4 and WT1 contributed to SOX6‐induced cellular senescence in cervical cancer by synergistically activating the ATF2–TGFβ2–Smad2/3 signaling pathway. However, other factors may also be involved in these cascades, and the above pathways may not be the only mechanism of the SOX6‐induced cellular senescence in cervical cancer cells, which requires further exploration. Furthermore, we found that the activation of the ATF2–TGFβ2–Smad2/3 pathway mediated the SOX6‐induced senescence of CC cells, which contributed to resistance to cisplatin treatment. Senolytics could be used to enhance sensitivity to cisplatin treatment by inducing apoptosis of the SOX6‐induced senescent CC cells. This study first finds that SOX6 inhibits the proliferation of CC cells by inducing cellular senescence and uncovers its potential mechanism. More importantly, senolytics can be used to eliminate the SOX6‐induced senescent CC cells and thus improve the efficacy of cisplatin in treating CC. Therefore, the SOX6‐induced cellular senescence is a promising therapeutic target of CC, and the combination of SOX6‐induced cellular senescence and senolytics treatment is a new strategy to overcome cisplatin resistance.

## Conflict of interest

The authors declare no conflict of interest.

## Author contributions

HZ, ML, and SS were involved in conceptualization, formal analysis, investigation, methodology, analysis of data, data interpretation, visualization, and writing—original draft. HH, XY, ZL, YS, and QX were involved in methodology, analysis of data, and data interpretation. TL, LX, and FL were involved in methodology, data interpretation, writing—editing, and review. JW was involved in conceptualization, investigation, methodology, data interpretation, resources, funding acquisition, supervision, project administration, writing—editing, and review. All authors approved the final manuscript.

## Supporting information


**Fig. S1.** The correlation between *SOX6* mRNA and its gene copy number.
**Fig. S2.** The levels of SOX6 protein in cervical cancer tissues were lower than normal tissues.
**Fig. S3.** SOX6 inhibits cellular proliferation in HeLa‐HA‐SOX6‐tet cells.
**Fig. S4.** HPV18 E6 and E7 could inhibit SOX6 protein expression in cervical cancer cells.
**Fig. S5.** SOX6 induces autophagic senescence in cervical cancer cells.
**Fig. S6.** Quantification of relative protein levels presented in Figs 3H and 4A by gray value analyses.
**Fig. S7.** TGFβ2 is involved in the SOX6‐induced senescence of cervical cancer cells.
**Fig. S8.** *TGFB2* gene is not the direct target gene of SOX6.
**Fig. S9.** Quantification of relative protein levels presented in Fig. 5D,F by gray value analyses.
**Fig. S10.** MAP4K4 (JNK/ERK/p38)–ATF2 pathway mediates the SOX6‐induced senescence of cervical cancer cells.
**Fig. S11.** Inhibiting MAP4K4 by PF‐06260933 could induce apoptosis in HeLa‐HA‐SOX6‐tet cells.
**Fig. S12.** WT1 mediates SOX6 to promote ATF2 expression.
**Fig. S13.** Senolytics induce apoptosis of the SOX6‐mediated cisplatin‐resistant cervical cancer cells.

**Table S1.** The primer sequences used in vector construction.
**Table S2.** The primer sequences used for qPCR.
**Table S3.** The antibodies used in immunofluorescence staining, IHC and western blot analyses.

## Data Availability

The data that support the findings of this study are available from the corresponding author upon reasonable request.
