# Supplementary material for: MAP4K4 and WT1 mediate SOX6‐induced cellular senescence by synergistically activating the ATF2–TGFβ2–Smad2/3 signaling pathway in cervical cancer
Source: Mol Oncol. 2024 Feb 21;18(5):1327–46. doi: 10.1002/1878-0261.13613 (PMC11076992; doi:10.1002/1878-0261.13613)
Supplement: Supplementary file 1 — Fig. S1. The correlation between SOX6 mRNA and its gene copy number. Fig. S2. The levels of SOX6 protein in cervical cancer tissues were lower than normal tissues. Fig. S3. SOX6 inhibits cellular proliferation in HeLa‐HA‐SOX6‐tet cells. Fig. S4. HPV18 E6 and E7 could inhibit SOX6 protein expression in cervical cancer cells. Fig. S5. SOX6 induces autophagic senescence in cervical cancer cells. Fig. S6. Quantification of relative protein levels presented in Figs 3H and 4A by gray value analyses. Fig. S7. TGFβ2 is involved in the SOX6‐induced senescence of cervical cancer cells. Fig. S8. TGFB2 gene is not the direct target gene of SOX6. Fig. S9. Quantification of relative protein levels presented in Fig. 5D,F by gray value analyses. Fig. S10. MAP4K4 (JNK/ERK/p38)–ATF2 pathway mediates the SOX6‐induced senescence of cervical cancer cells. Fig. S11. Inhibiting MAP4K4 by PF‐06260933 could induce apoptosis in HeLa‐HA‐SOX6‐tet cells. Fig. S12. WT1 mediates SOX6 to promote ATF2 expression. Fig. S13. Senolytics induce apoptosis of the SOX6‐mediated cisplatin‐resistant cervical cancer cells. [file MOL2-18-1327-s002.docx]

**Supplementary figures**

**Supplementary figure 1**


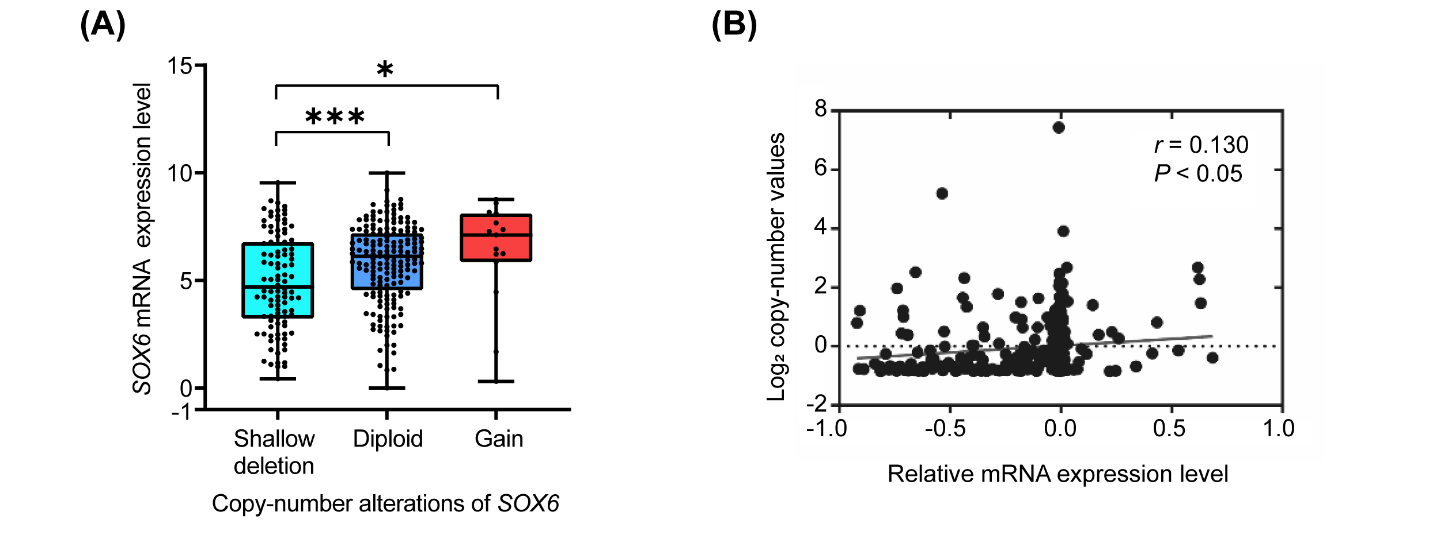


Figure S1. The correlation between *SOX6* mRNA and its gene copy number. (A) The levels of *SOX6* mRNA in 293 cervical cancer samples with different copy number of *SOX6* gene were analyzed in cBioPortal database. The number of samples with shallow depleted, diploid and gained *SOX6* gene was 102, 172 and 15, respectively. **P* < 0.05, ****P* < 0.001, Student’s *t*-test. (B) The correlation between *SOX6* mRNA level and its gene copy number in 293 cervical cancer samples from cBioPortal database was analyzed by Spearman correlation analysis.

**Supplementary figure 2**

**
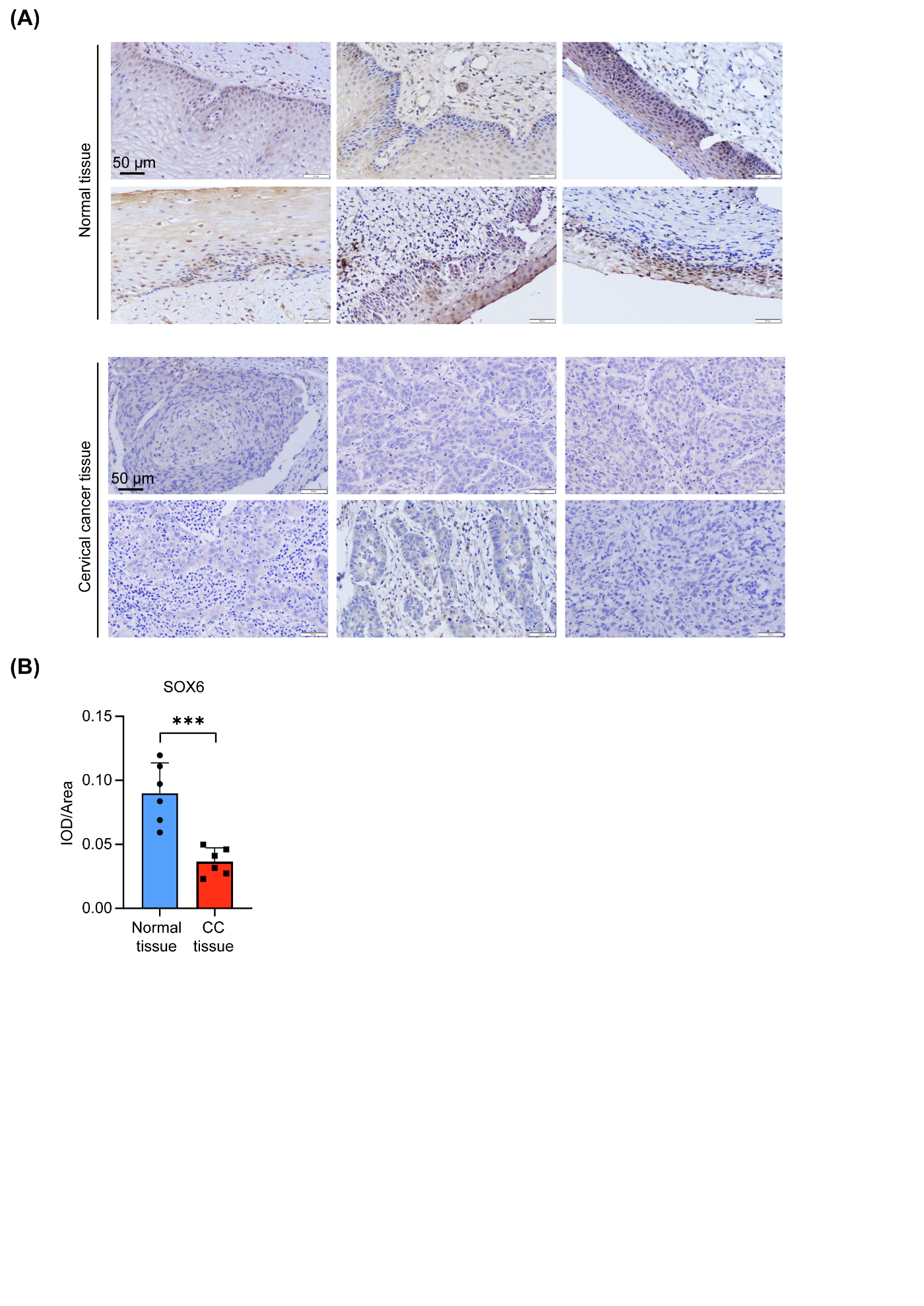
**

Figure S2. The levels of SOX6 protein in cervical cancer tissues were lower than normal tissues. (A) The levels of SOX6 protein in cervical cancer tissues and normal tissues in tissue microarray. (B) Relative expression of SOX6 protein was analyzed by calculating integrated optical density per stained area (IOD/Area). ****P* < 0.001, Student’s *t*-test, two tails. CC, cervical cancer.

**Supplementary figure 3**

**
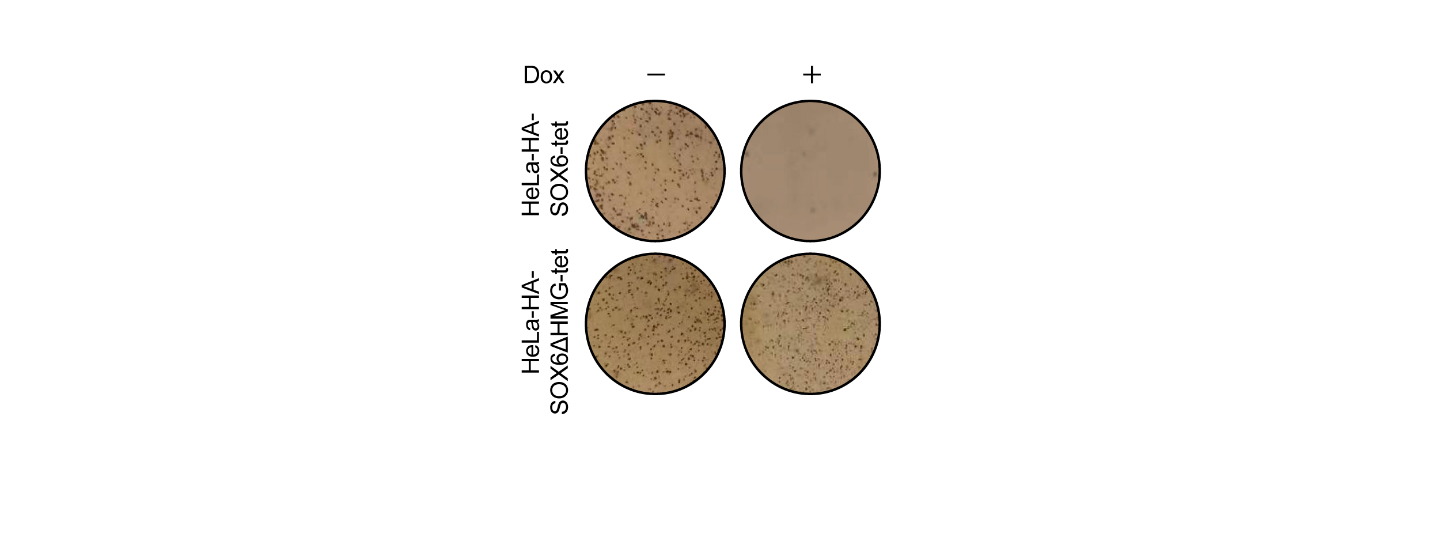
**

Figure S3. SOX6 inhibits cellular proliferation in HeLa-HA-SOX6-tet cells. HeLa-HA-SOX6-tet and HeLa-HA-SOX6ΔHMG-tet cells were plated into soft agar plates and were treated with Dox (2 μg/ml) or solvent control. After incubating for 2 weeks, the cell colonies were stained with MTT and were observed under natural light. Dox, doxycycline.

**Supplementary figure 4**

**
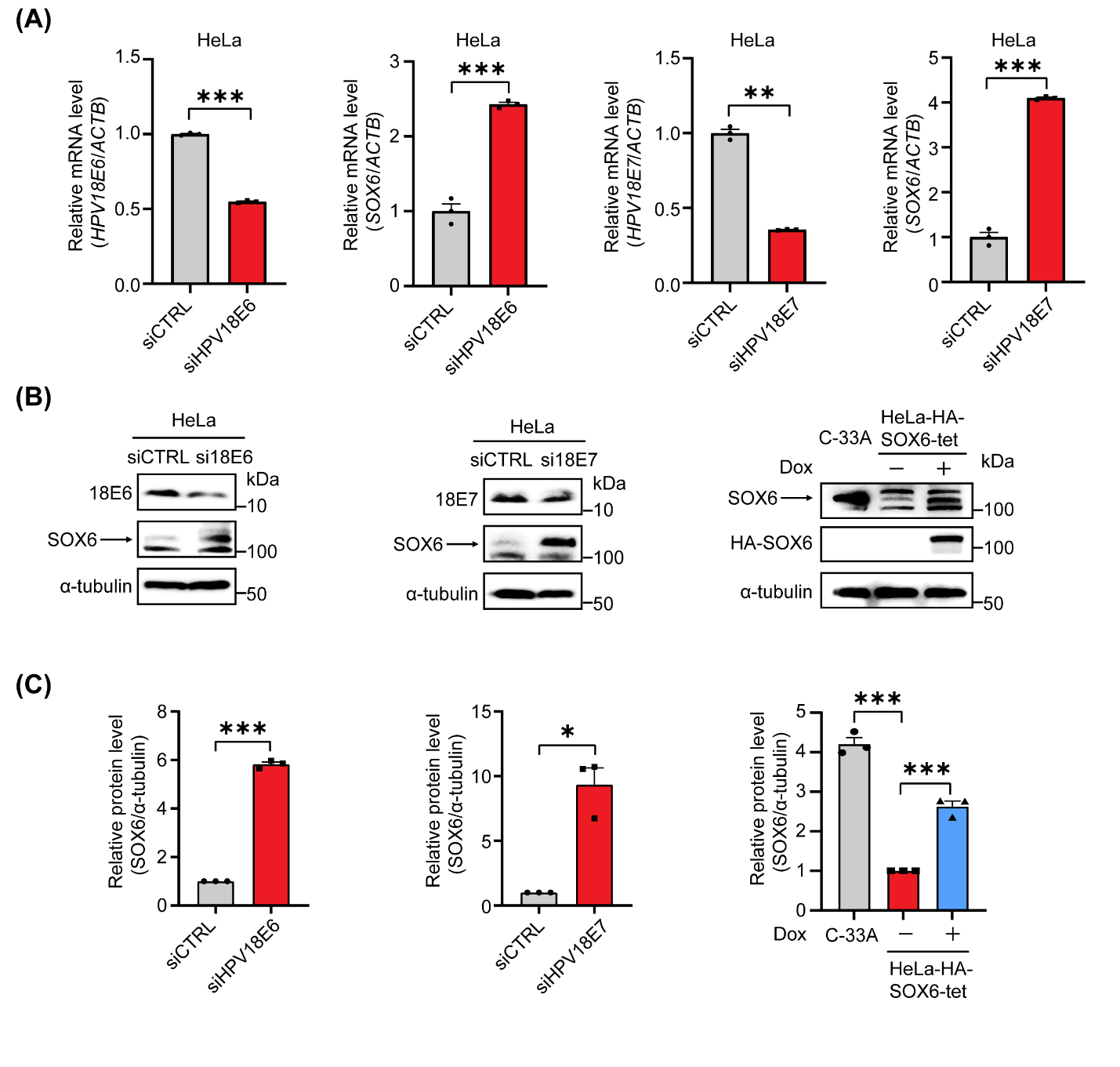
**

Figure S4. HPV18 E6 and E7 could inhibit SOX6 protein expression in cervical cancer cells. (A) HeLa cells were transfected with HPV18 E6-specific and HPV18 E7-specific siRNA or control siRNA (siCTRL). The mRNA levels were analyzed by RT-qPCR. (B) HeLa cells were transfected with HPV18 E6-specific and HPV18 E7-specific siRNA or control siRNA (siCTRL) and the protein levels of SOX6 were analyzed by Western blot. Besides, the protein levels of SOX6 and HA-SOX6 in C-33A and HeLa-HASOX6-tet cells treated with Dox (2 μg/mL) or solvent control were analyzed by Western blot. (C) The quantification on the relative protein levels of SOX6 in Fig. S4B were performed. Data were shown as mean ± s.e.m. of three independent experiments. **P* < 0.05, ***P* < 0.01, ****P* < 0.001, Student’s *t*-test. Dox, doxycycline.

**Supplementary figure 5**


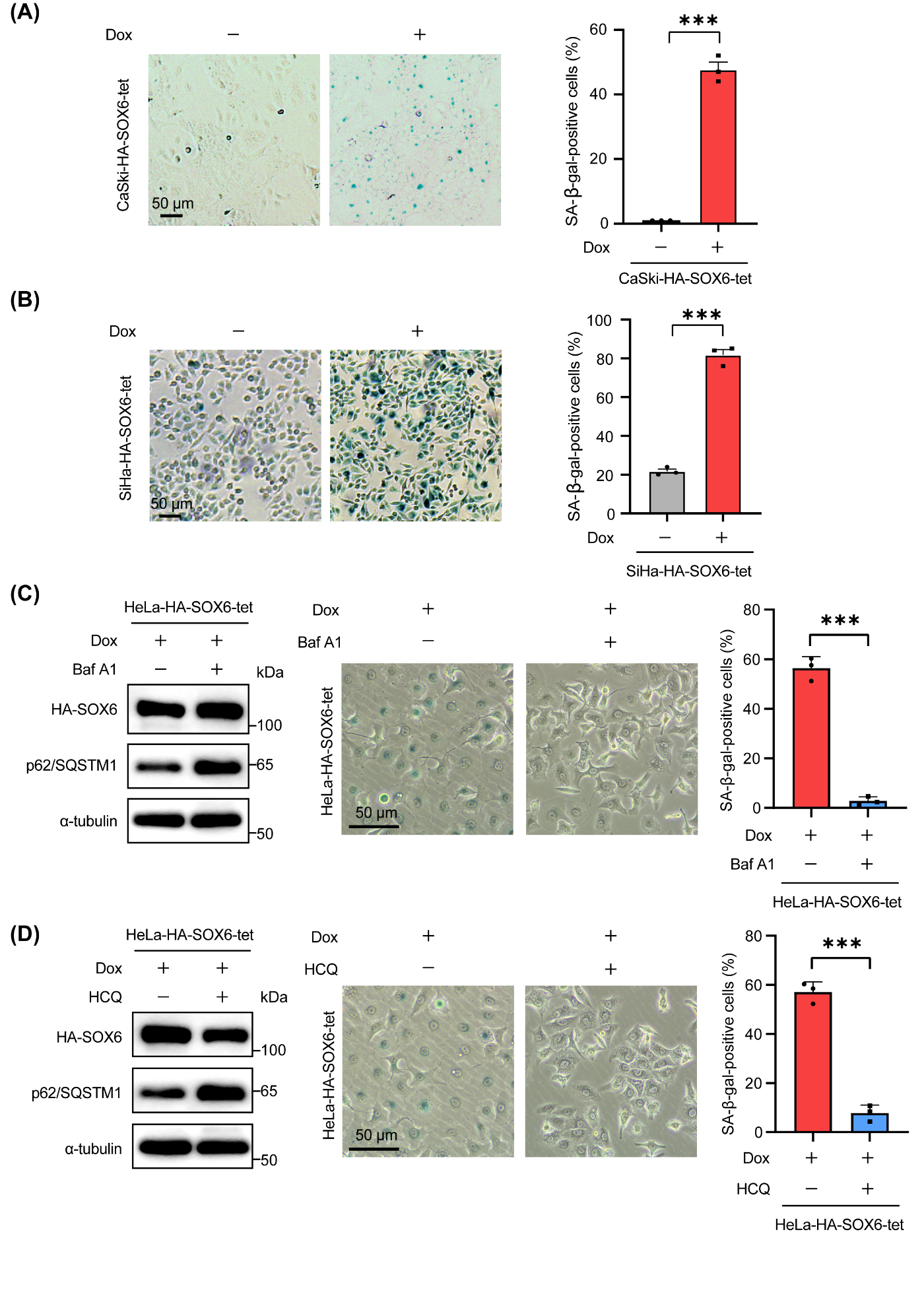


Figure S5. SOX6 induces autophagic senescence in cervical cancer cells. (A) CaSki-HA-SOX6-tet cells and (B) SiHa-HA-SOX6-tet cells were treated with Dox (2 μg/mL) or solvent control for 4 days. The senescent cells were detected by SA-β-gal staining, and the percentage of SA-β-gal-positive cells was analyzed at three fields. (C) HeLa-HA-SOX6-tet cells were treated with Dox (2 μg/mL) and Baf A1 (10 nM), (D) or hydroxychloroquine (HCQ) (20 μM) for 4 days. The protein levels of HA-SOX6 and p62/SQSTM1 were detected by Western blot. α-tubulin was used as the internal control. The senescent cells were detected by SA-β-gal staining, and the percentage of SA-β-gal-positive cells was analyzed at three fields. Data were shown as mean ± s.e.m. of three independent experiments. ****P* < 0.001, Student’s *t*-test. Dox, doxycycline.

**Supplementary figure 6**

**
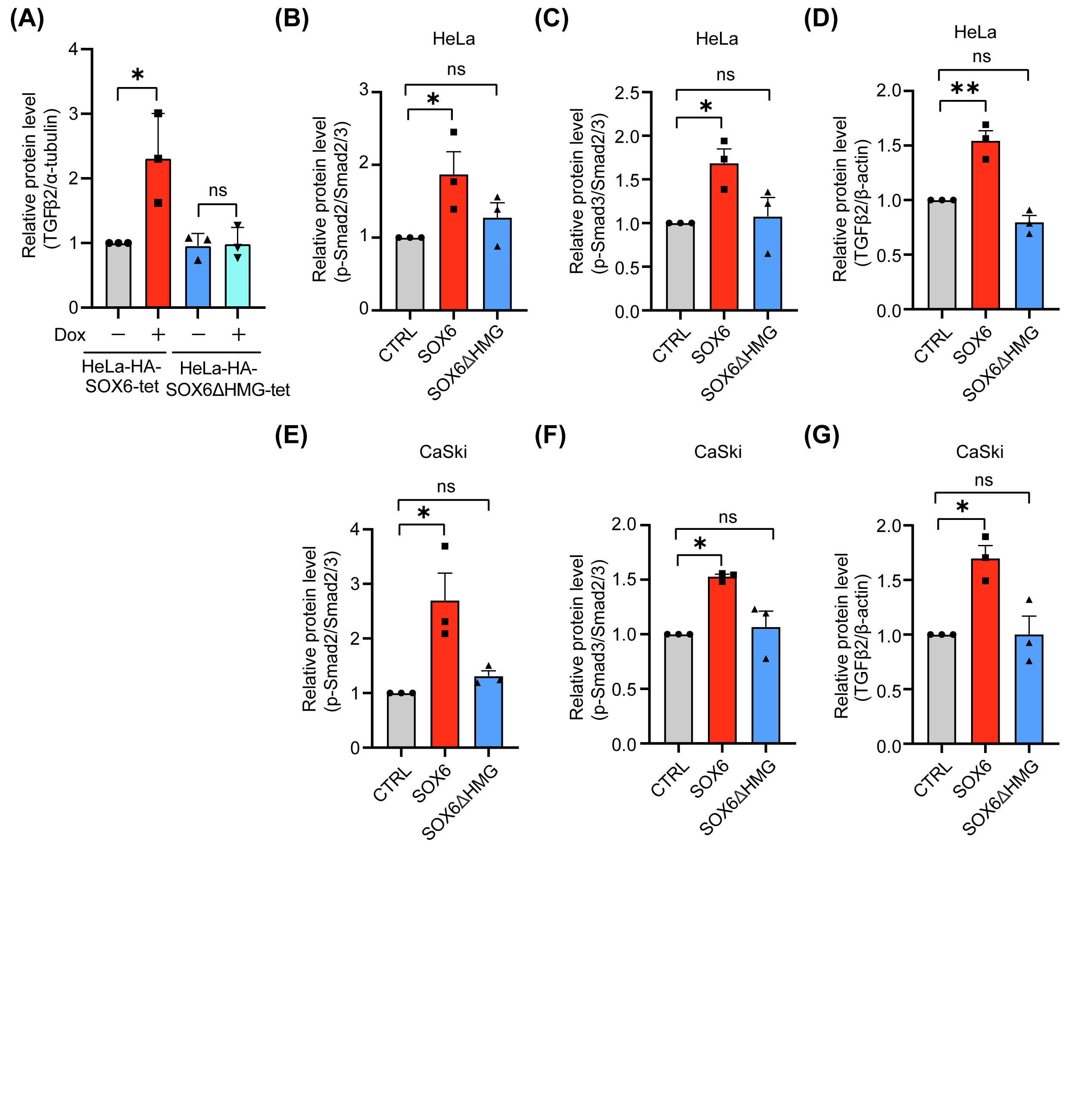
**

Figure S6. Quantification of relative protein levels presented in Fig. 3H and 4A by gray value analyses. (A) Quantification on the relative levels of TGFβ2 in HeLa-HA-SOX6-tet and HeLa-HA-SOX6ΔHMG-tet cells treated with Dox (2 μg/ml) or solvent control. (B) Quantification on the relative protein levels of p-Smad2, p-Smad3 (C) and TGFβ2 (D) in HeLa cells transfected with plex-HA-SOX6, plex-HA-SOX6ΔHMG or vector control (CTRL) plasmids. (E) Quantification on the relative protein levels of p-Smad2, p-Smad3 (F) and TGFβ2 (G) in CaSki cells transfected with plex-HA-SOX6, plex-HA-SOX6ΔHMG or vector control (CTRL) plasmids. Data were shown as mean ± s.e.m. of three independent experiments. **P* < 0.05, ***P* < 0.01, ns non-significant, Student’s *t*-test. Dox, doxycycline.

**Supplementary figure 7**

**
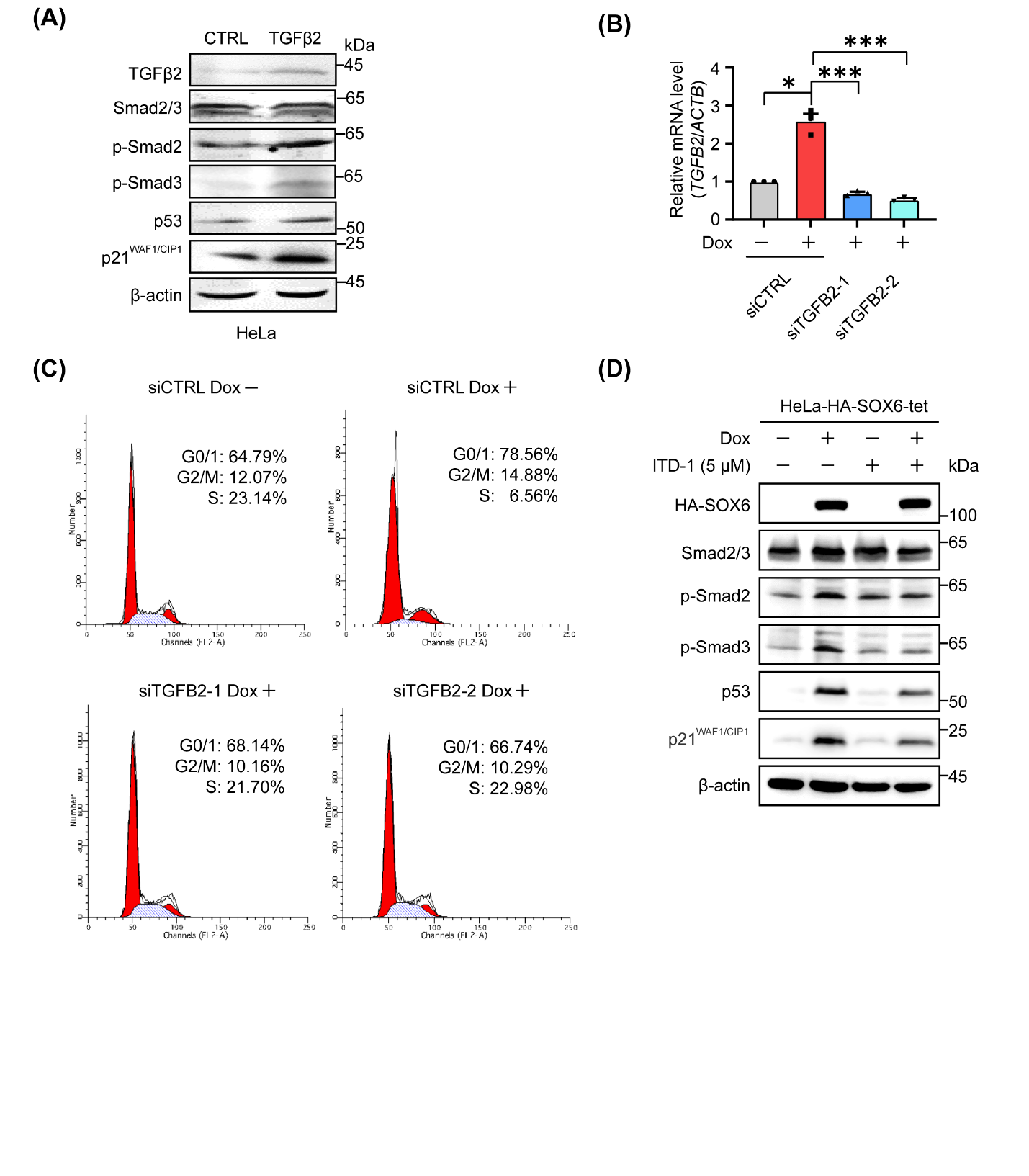
**

Figure S7. TGFβ2 is involved in the SOX6-induced senescence of cervical cancer cells. (A) HeLa cells were transfected with pCDH-TGFβ2 plasmid or vector control (CTRL), the levels of TGFβ2, Smad2/3, p-Smad2/3, p53, and p21^WAF1/CIP1^ proteins were detected by Western blot. β-actin was used as the internal control. (B) HeLa-HA-SOX6-tet cells were transfected with siTGFB2-1, siTGFB2-2 or siCTRL and were treated with Dox (2 μg/mL) or solvent control for 4 days. The level of *TGFB2* mRNA was detected by RT-qPCR. *ACTB* mRNA was used as the internal control. (C) The cell cycle of HeLa-HA-SOX6-tet cells was measured by flow cytometry analyses. (D) HeLa-HA-SOX6-tet cells were treated with Dox (2 μg/mL) or solvent control and ITD-1 (5 μM). The corresponding protein levels were detected by Western blot. β-actin protein was used as the internal controls. Data were shown as mean ± s.e.m. of three independent experiments. **P* < 0.05, ****P* < 0.001, Student’s *t*-test. Dox, doxycycline.

**Supplementary figure 8**


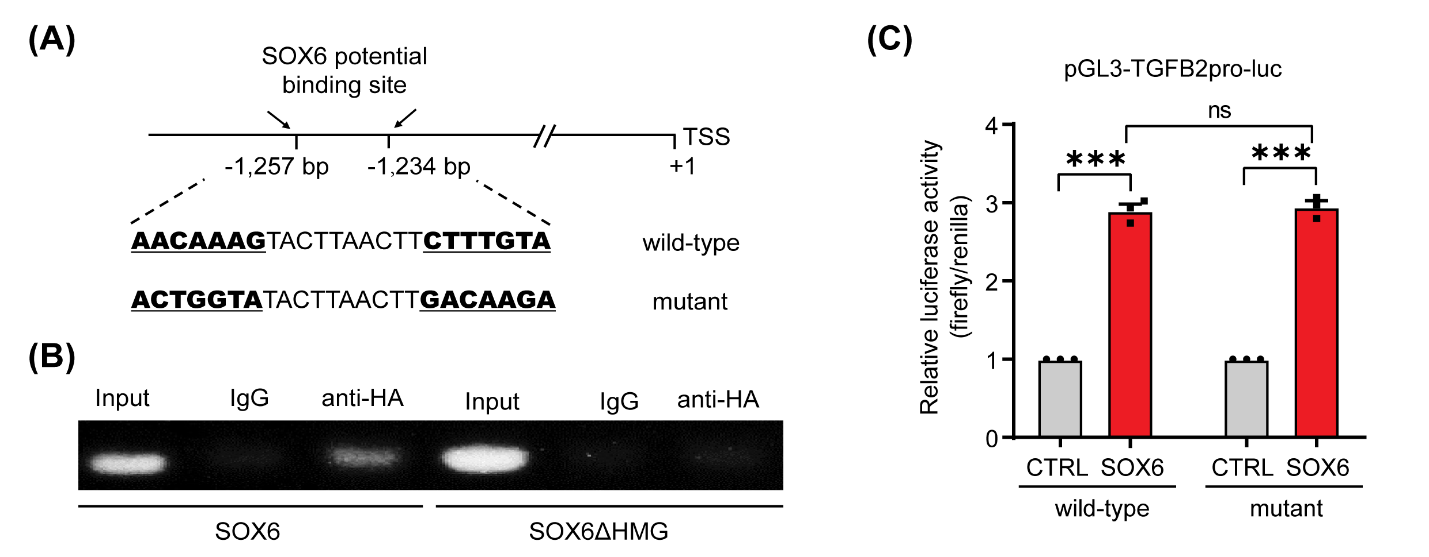


Figure S8. *TGFB2* gene is not the direct target gene of SOX6. (A) The wild-type and mutant sequences of SOX6 potential binding site at 1,257-1,234 bp upstream of *TGFB2* gene TSS. (B) The plex-HA-SOX6 or plex-HA-SOX6ΔHMG plasmid was transfected into HeLa cells. HA-labeled SOX6 and SOX6ΔHMG protein were immunoprecipitated by anti-HA antibody, respectively. The potential binding region of *TGFB2* gene promoter was amplified by PCR, and then the PCR products were detected by 1.5% agarose gel electrophoresis. (C) The pGL3-TGFB2pro-luc (wild-type) or pGL3-TGFB2pro-mutant (SOX6)-luc (mutant) plasmid, PRL-TK and plex-HA-SOX6 or vector control (CTRL) were co-transfected into HeLa cells. Dual-luciferase assay was performed to detect the transcriptional activities of the wild-type and SOX6 binding site mutant *TGFB2* gene promoters. Data were shown as mean ± s.e.m. of three independent experiments. ****P* < 0.001, ns non-significant, Student’s *t*-test. TSS, transcription start site.

**Supplementary figure 9**

**
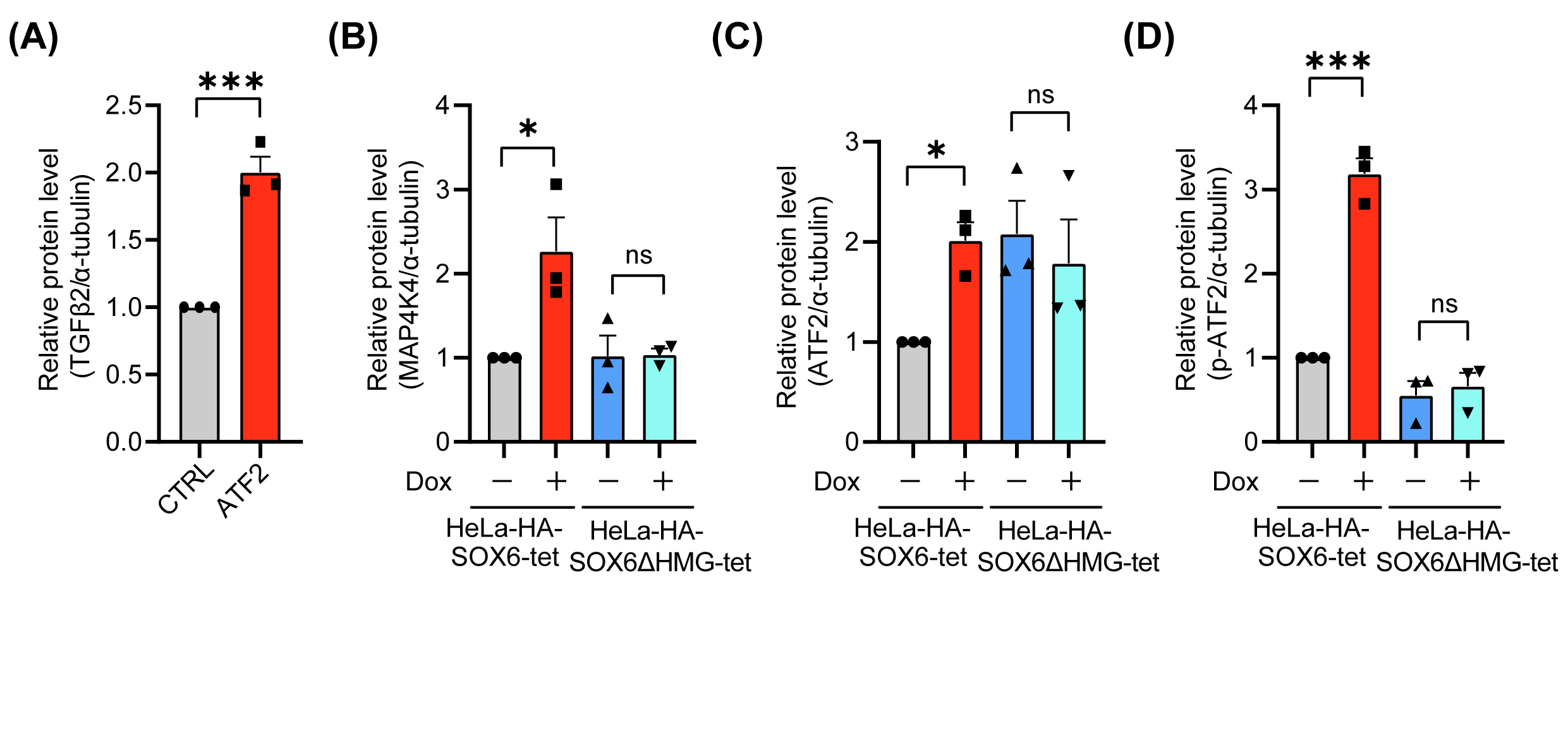
**

Figure S9. Quantification of relative protein levels presented in Fig. 5D and 5F by gray value analyses. (A) Quantification on the relative levels of TGFβ2 in HeLa cells transfected with pCDH-ATF2 or vector control (CTRL) plasmids. (B) Quantification on the relative levels of MAP4K4, ATF2 (C) and p-ATF2 (D) in HeLa-HA-SOX6-tet and HeLa-HA-SOX6ΔHMG-tet cells treated with Dox (2 μg/ml) or solvent control. Data were shown as mean ± s.e.m. of three independent experiments. **P* < 0.05, ****P* < 0.001, ns non-significant, Student’s *t*-test. Dox, doxycycline.

**Supplementary figure 10**


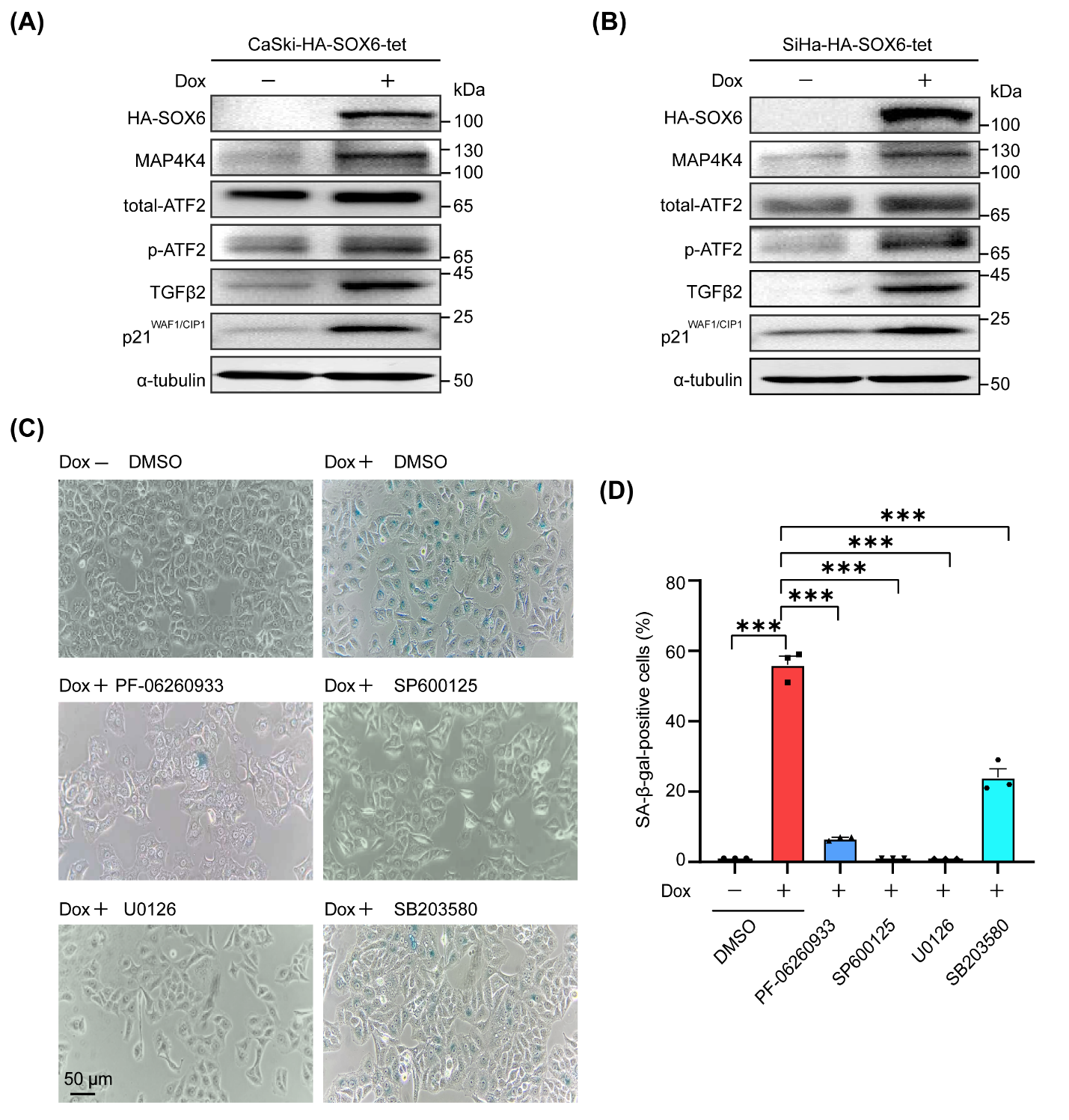


Figure S10. MAP4K4 (JNK/ERK/p38)–ATF2 pathway mediates the SOX6-induced senescence of cervical cancer cells. (A) CaSki-HA-SOX6-tet and (B) SiHa-HA-SOX6-tet cells were treated with Dox (2 μg/mL) or solvent control. The protein levels of MAP4K4, total-ATF2, p-ATF2, TGFβ2 and p21^WAF1/CIP1^ were detected by Western blot. α-tubulin was used as the internal control. (C) HeLa-HA-SOX6-tet cells were co-treated with Dox (2 μg/mL) or solvent control and PF-06260933 (10 μM), U0126 (10 μM), SP600125 (10 μM) or SB203580 (10 μM) for 4 days. The senescent cells were detected by SA-β-gal staining. (D) The percentage of SA-β-gal-positive cells was analyzed at three fields. Data were shown as mean ± s.e.m. of three independent experiments. ****P* < 0.001, Student’s *t*-test. Dox, doxycycline.

**Supplementary figure 11**

**
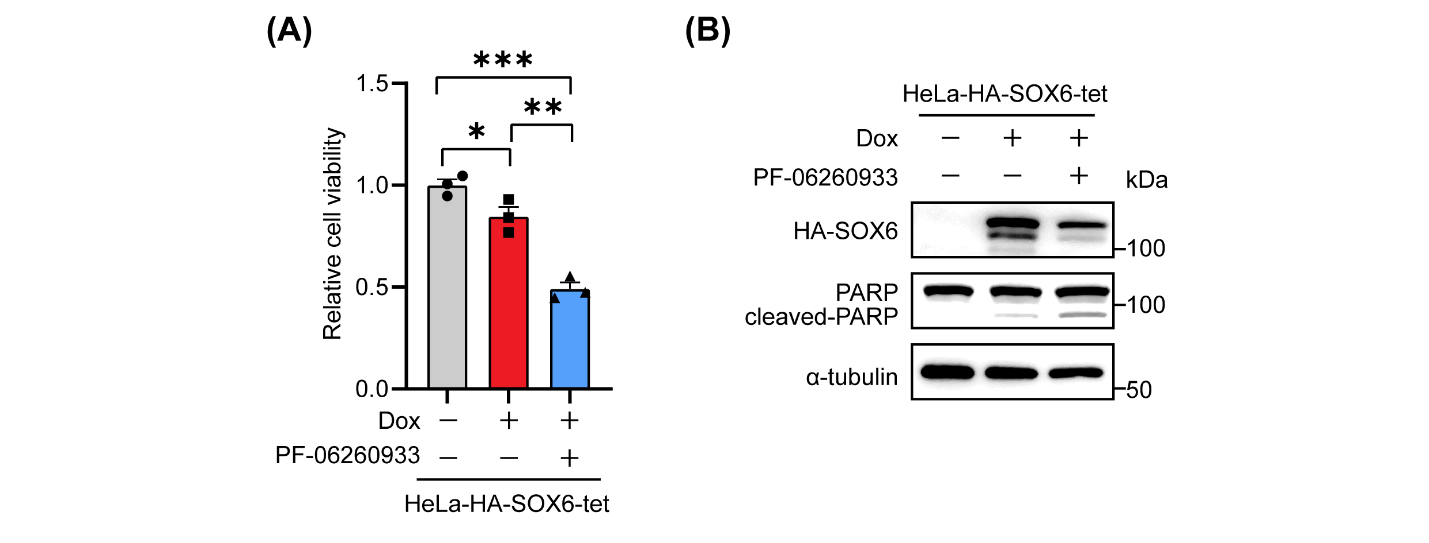
**

Figure S11. Inhibiting MAP4K4 by PF-06260933 could induce apoptosis in HeLa-HA-SOX6-tet cells. HeLa-HA-SOX6-tet cells were treated with or without Dox (2 μg/ml) combined with MAP4K4 inhibitor, PF-06260933 (10 μM). (A) The cell viability was analyzed by CCK-8 assays after 4 days treatment. (B) The protein levels of HA-SOX6, PARP and cleaved-PARP were detected by Western blot. α-tubulin was used as the internal control. Data were shown as mean ± s.e.m. of three independent experiments. **P* < 0.05, ***P* < 0.01, ****P* < 0.001, Student’s *t*-test.­­­ Dox, doxycycline.

**Supplementary figure 12**


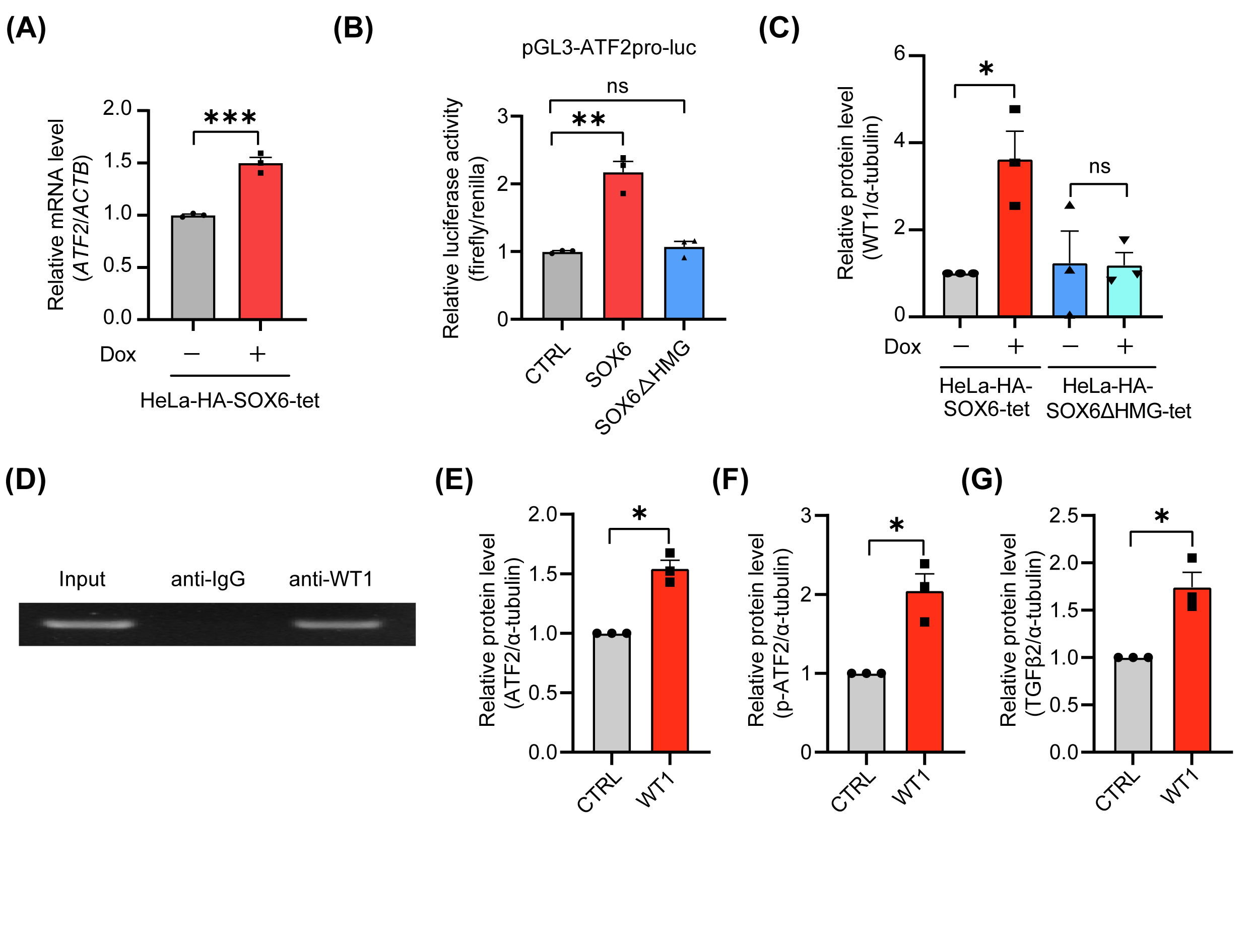


Figure S12. WT1 mediates SOX6 to promote ATF2 expression. (A) HeLa-HA-SOX6-tet cells were treated with Dox (2 μg/mL) or solvent control. The level of *ATF2* mRNA was detected by RT-qPCR (SYBR green). (B) The pGL3-ATF2pro-luc, pRL-TK (Renilla), and pLex-HA-SOX6, pLex-HA-SOX6ΔHMG or vector control (CTRL) plasmids were co-transfected into HeLa cells. The transcriptional activity of *ATF2* gene promoter was detected by dual-luciferase assay. (C) Quantification on the relative levels of WT1 in HeLa-HA-SOX6-tet and HeLa-HA-SOX6ΔHMG-tet cells treated with Dox (2 μg/ml) or solvent control. (D) The pCDH-flag-WT1 plasmid was transfected into HeLa cells. WT1 protein was immunoprecipitated by anti-WT1 antibody, and the potential binding region of *ATF2* gene promoter was amplified by PCR. The PCR products were detected by 1.5% agarose gel electrophoresis. (E) Quantification on the relative levels of ATF2, p-ATF2 (F) and TGFβ2 (G) in HeLa cells transfected with pCDH-WT1 or vector control (CTRL) plasmids. Data were shown as mean ± s.e.m. of three independent experiments. **P* < 0.05, ***P* < 0.01, ****P* < 0.001, ns non-significant, Student’s *t*-test.­­­ Dox, doxycycline.

**Supplementary figure 13**


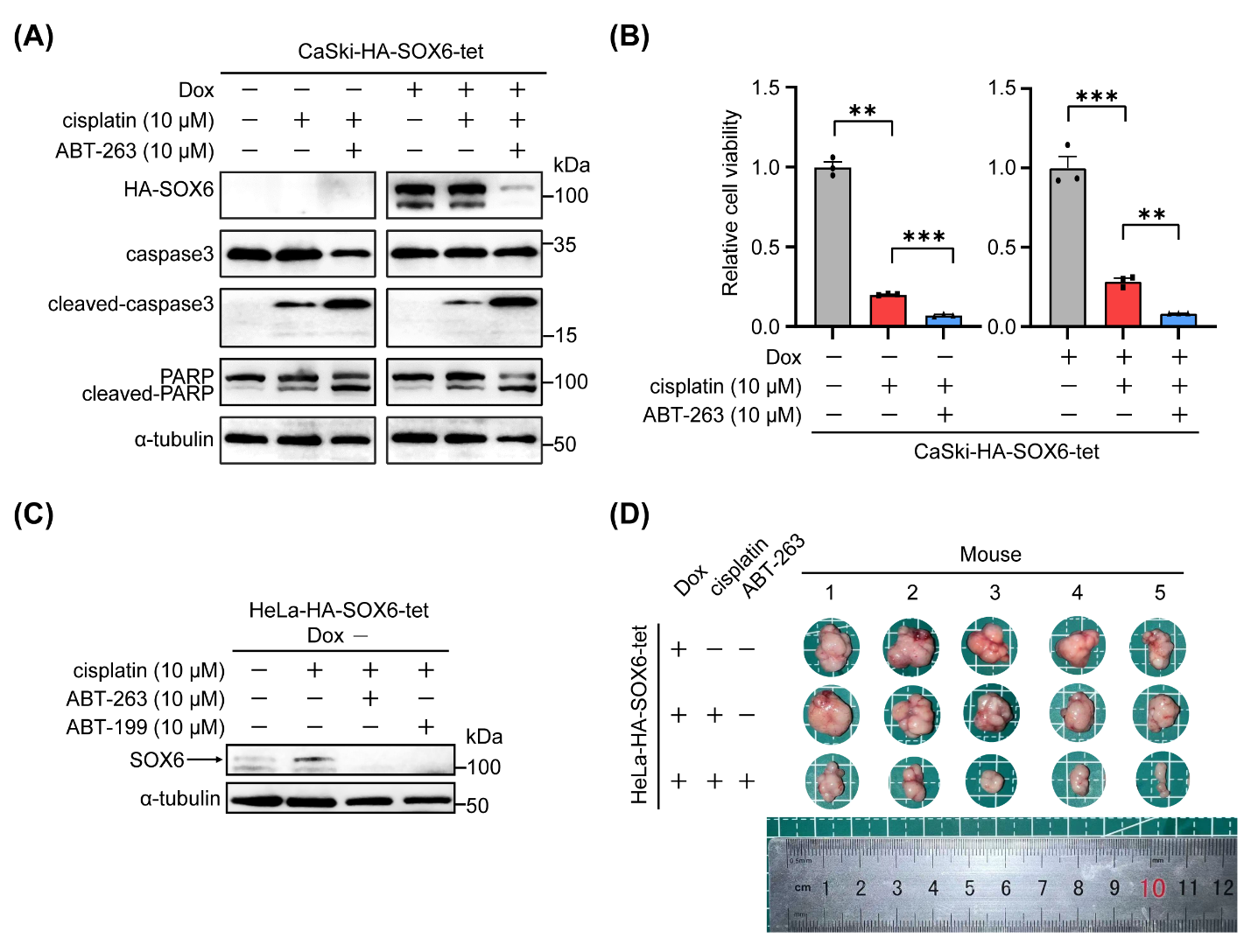


Figure S13. Senolytics induce apoptosis of the SOX6-mediated cisplatin resistant cervical cancer cells. (A) CaSki-HA-SOX6-tet cells were pretreated with Dox (2 μg/mL) or solvent control for 4 days, and then were treated with cisplatin (10 μM) and ABT-263 (5 μM) or solvent control for another 2 days. The levels of apoptosis-related proteins were detected by Western blot. (B) The cell viability was assessed by CCK-8 assays. (C) HeLa-HA-SOX6-tet cells were pretreated with Dox (2 μg/mL) or solvent control for 4 days, and then were treated with cisplatin (10 μM) and ABT-263 (10 μM), ABT-199 (10 μM) or solvent control for another 2 days. The level of endogenous SOX6 protein was detected by Western blot. (D) The tumor blocks in the nude mice of xenograft tumor experiment. α-tubulin was used as the internal control. Data were shown as mean ± s.e.m. of three independent experiments. ***P* < 0.01, ****P* < 0.001, Student’s *t*-test. Dox, doxycycline.
