# Supplementary material for: MAP4K4 and WT1 mediate SOX6‐induced cellular senescence by synergistically activating the ATF2–TGFβ2–Smad2/3 signaling pathway in cervical cancer
Source: Mol Oncol. 2024 Feb 21;18(5):1327–46. doi: 10.1002/1878-0261.13613 (PMC11076992; doi:10.1002/1878-0261.13613)
Supplement: Supplementary file 2 — Table S1. The primer sequences used in vector construction. Table S2. The primer sequences used for qPCR. Table S3. The antibodies used in immunofluorescence staining, IHC and western blot analyses. [file MOL2-18-1327-s001.docx]

**Supplementary tables**

**Table S1. The primer sequences used in vector construction.**

| Primer name | Primer sequences (5’-3’) |
| --- | --- |
| pGL3-TGFB2pro-luc | F: GGGGTACCGTTAAAAGAGTGAAAAGGTTTTC  R: CCGCTCGAGACGATCTTGCCGGGGAGGGGAG |
| pGL3-TGFB2pro-mutant (SOX6)-luc | F: CTACCACTTACTGTGGTCTCGACTGGTATACTTAACTTGACAAGACCTCAGTCTCCTCATCTGCCA  R: TGGCAGATGAGGAGACTGAGGTCTTGTCAAGTTAAGTATACCAGTCGAGACCACAGTAAGTGGTAG |
| pGL3-TGFB2pro-mutant (ATF2)-luc | F: GACTTCTGACTGTAATCCTAGTCTTCGCAGCTGTTGAAGGCAGACACGTGGT  R: ACCACGTGTCTGCCTTCAACAGCTGCGAAGACTAGGATTACAGTCAGAAGTC |
| pGL3-TGFB2pro-mutant (LEF1)-luc | F: ACTGTAATCCTAGCACGTCAAGGCACCTAAGGCAGACACGTGGTTCAG  R: CTGAACCACGTGTCTGCCTTAGGTGCCTTGACGTGCTAGGATTACAGT |
| pGL3-TGFB2pro-mutant (c-Myc)-luc | F: CGTCACTTTGTTGAAGGCAGATCATTAGTTCAGAGAGAACTTATAAAT  R: ATTTATAAGTTCTCTCTGAACTAATGATCTGCCTTCAACAAAGTGACG |
| pGL3-TGFB2pro-mutant (MAZ)-luc | F: GTGGTTCAGAGAGAACTTATAAATCCGTTACGTAAGGCAAGATCGTAAGCTTGGCATTCC  R: GGAATGCCAAGCTTACGATCTTGCCTTACGTAACGGATTTATAAGTTCTCTCTGAACCAC |
| pGL3-WT1pro-luc | F: GGGGTACCACTGGCTTCCGCCTCCTGGT  R: CGACGCGTAAATAAGAGGGGCCGGCGG |
| pGL3-WT1pro-mutant (SOX6)-luc | F: CAAGAGCCAGACTCAAGGGTGTGCGACAAGGGTATACGCTTACGCTAAGCTTGACTGAGTTCTTTCT  R: AGAAAGAACTCAGTCAAGCTTAGCGTAAGCGTATACCCTTGTCGCACACCCTTGAGTCTGGCTCTTG |
| pGL3-ATF2pro-luc | F: GGGGTACCCCTGCATCATGGAACAAGACAGGC  R: CGACGCGTCGCGAGATCGGACTGACCAACC |
| pCDH-flag-TGFB2 | F: CGGGATCCCGGCCACCATGGACTACAAAGACCATGACGGTGATTATAAAGATCATGACATCGACTACAAGGATGACGATGACAAGATGCACTACTGTGTGCTGAGC  R: GCTCTAGAGCTTAGCTGCATTTGCAAGACTTTAC |
| pCDH-flag-ATF2 | F: CGCGGATCCGCCGCCACCATGGACTACAAAGACCATGACGGTGATTATAAAGATCATGACATCGACTACAAGGATGACGATGACAAGATGAAATTCAAGTTACATGTGAATT  R: ATAAGAATGCGGCCGCTCAACTTCCTGAGGGCTGTGA |
| pCDH-flag-WT1 | F: ATTAAGAATGCGGCCGCATGGACTACAAAGACCATGACGGTGATTATAAAGATCATGACATCGACTACAAGGATGACGATGACAAGATGGACTTCCTCTTGCTGCAGG  R: GCTCTAGATCAAAGCGCCAGCTGGAGTT |
| pCDH-HPV18E6-flag | F: GGAATTCGCCACCATGGCGCGCTTTGAGGAT  R: ATAAGAATGCGGCCGCTTACTTGTCATCGTCATCCTTGTAGTCGATGTCATGATCTTTATAATCACCGTCATGGTCTTTGTAGTCTACTTGTGTTTCTCTGCGTCG |
| pCDH-HPV18E7-flag | F: GGAATTCGCCACCATGCATGGACCTAAGGCAAC  R: ATAAGAATGCGGCCGCTTACTTGTCATCGTCATCCTTGTAGTCGATGTCATGATCTTTATAATCACCGTCATGGTCTTTGTAGTCCTGCTGGGATGCACACC |

F: forward, R: reverse.

**Table S2. The primer sequences used for qPCR.**

| Gene name | Primer sequences (5’-3’) |
| --- | --- |
| *WT1* | F: ACAGATGCACAGCCGGAAGC  R: GGTGGTCGGAACGGGAGAA |
| *ATF2* | F: ATGGTAGCGGATTGGTTA  R: TCGGCACTGAAATGTCTT |
| *TGFB2* | F: AAGTCATACCACCTTTCCGATTG  R: ACGGCACAGGGATTTCTTCTA |
| *SOX6* | F: GGACAGCGTTCTGTCATCTC  R: CTCTTGTTCAGTCCGAGTCA |
| *ACTB* | F: CTACAGCTTCACCACCACGG  R: TCAGGCAGCTCGTAGCTCTTC |

F: forward, R: reverse.

**Table S3. The antibodies used in immunofluorescence staining, IHC and Western blot analyses.**

| Antibody name | Manufacturer | Catalog No. | Application |
| --- | --- | --- | --- |
| anti-SOX6 | Abcam | ab30455 | WB, IF |
| anti-phospho-JNK | Abcam | ab124956 | WB |
| anti-p38 | Abcam | ab31828 | WB |
| anti-p16^INK4a^ | Abcam | ab108349 | WB |
| anti-MAP4K4 | Santa Cruz Biotechnology | sc-100445 | WB |
| anti-JNK | Santa Cruz Biotechnology | sc-7345 | WB |
| anti-phospho-p38 | Santa Cruz Biotechnology | sc-166182 | WB |
| anti-TGFβ2 | Santa Cruz Biotechnology | sc-374658 | WB |
| anti-Rb | Santa Cruz Biotechnology | sc-102 | WB |
| anti-β-actin | Santa Cruz Biotechnology | sc-1616 | WB |
| anti-ERK | Cell Signaling Technology | 9102S | WB |
| anti-phospho-ERK | Cell Signaling Technology | 4370S | WB |
| anti-WT1 | Cell Signaling Technology | 83535S | WB |
| anti-ATF2 | Cell Signaling Technology | 35031S | WB |
| anti-phospho-ATF2 | Cell Signaling Technology | 27934S | WB |
| anti-Smad2/3 | Cell Signaling Technology | 8685T | WB |
| anti-phospho-Smad2 | Cell Signaling Technology | 3108S | WB |
| anti-phospho-Smad3 | Cell Signaling Technology | 9520S | WB |
| anti-phospho-Rb | Cell Signaling Technology | 9301S | WB |
| anti-LC3B | Cell Signaling Technology | 2775S | WB |
| anti-PARP | Cell Signaling Technology | 9542S | WB |
| anti-Bax | Cell Signaling Technology | 5023S | WB |
| anti-Bcl-2 | Cell Signaling Technology | 15071S | WB |
| anti-caspase 9 | Cell Signaling Technology | 9502S | WB |
| anti-caspase 3 | Cell Signaling Technology | 9662S | WB |
| anti-cleaved caspase 3 | Cell Signaling Technology | 9661S | WB |
| anti-Flag | Sigma-Aldrich | F1804 | WB |
| anti-HA | MBL | M180-3 | WB |
| anti-p53 | MBL | K0181-3 | WB |
| anti-p21^WAF1/CIP1^ | MBL | K0081-3 | WB |
| anti-α-tubulin | MBL | PM054 | WB |
| anti-Lamin B1 | MBL | PM064 | WB |
| HRP-linked anti-rabbit | Cell Signaling Technology | 7074S | WB |
| HRP-linked anti-mouse | Cell Signaling Technology | 7076S | WB |
| IRDye 680LT donkey anti-rabbit | LICOR | 926-68023 | WB |
| IRDye 800CW donkey anti-mouse | LICOR | 926-32212 | WB |
| anti-ki67 | Abcam | ab245113 | IF |
| FITC-conjugated goat anti-mouse | Zhongshan Golden Bridge | ZF-0312 | IF |
| FITC-conjugated goat anti-rabbit | Zhongshan Golden Bridge | ZF-0311 | IF |
| TRITC-conjugated goat anti-mouse | Zhongshan Golden Bridge | ZF-0313 | IF |
| TRITC-conjugated goat anti-rabbit | Zhongshan Golden Bridge | ZF-0316 | IF |
